# A Comprehensive Chemical–Biological Investigation of the Moderately Toxic Plant *Prospero autumnale*: Insights into Its Bioactive Potential Using In Vitro and In Vivo Models

**DOI:** 10.3390/toxins18070285

**Published:** 2026-06-30

**Authors:** Maroua Korichi, Ouanissa Smara, Lilya Harchaoui, Gilda D’Urso, Latifa Khattabi, Agostino Casapullo, Gianluigi Lauro, Maria Giovanna Chini, Giuseppe Bifulco, Alessio Cimmino, Hocine Dendougui, Wafa Zahnit, Marco Masi, Mahdi Belguidoum

**Affiliations:** 1 Laboratory of Valorization and Promotion of Saharan Resources (VPRS), Faculty of Mathematics and Matter Sciences, University of Ouargla, Ouargla 30000, Algeria; ouanissasm@gmail.com (O.S.); hocinedendougui@gmail.com (H.D.); belguidoum.mahdi@univ-ouargla.dz (M.B.); 2 Research Laboratory on Arid Zones (LRZA), Faculty of Biological Sciences, University of Sciences and Technology Houari Boumediene (USTHB), BP 32 El-Alia, Bab Ezzouar, Algiers 16111, Algeria; lilya.h@live.fr; 3 Department of Pharmacy, University of Salerno, Via Giovanni Paolo II 132, 84084 Fisciano, Italy; gidurso@unisa.it (G.D.); casapullo@unisa.it (A.C.); glauro@unisa.it (G.L.); bifulco@unisa.it (G.B.); 4 Biotechnology Research Centre, Constantine (CRBT), Ali Mendjli Nouvelle Ville UV 03, BP E73, Constantine 25016, Algeria; latifa.khattabi@umc.edu.dz; 5 Department of Biosciences and Territory, University of Molise, Contrada Fonte Lappone, 86090 Isernia, Italy; mariagiovanna.chini@unimol.it; 6 Department of Chemical Sciences, University of Naples Federico II, 80126 Naples, Italy; alessio.cimmino@unina.it; 7 Laboratoire de Chimie, Ingénierie Moléculaire et Nanostructures, Department of Chemistry, Faculty of Sciences, University of Ferhat ABBAS Setif 1, Setif 19000, Algeria; wafa.zahnit@univ-setif.dz; 8 Applied Sciences Faculty, University of Ouargla, Ouargla 30000, Algeria

**Keywords:** medicinal plants, phenolic compounds, LC–MS/MS analysis, acute toxicity, dose–response, antioxidant activity, anti-inflammatory activity

## Abstract

*Prospero autumnale* L. is a Mediterranean medicinal plant traditionally employed for inflammatory and neurological disorders. Nonetheless, its safety profile, toxicity, and application for treating inflammation and pain are yet to be comprehensively established. This investigation aimed to assess the bioactivity and toxicity of extracts derived from its aerial (AgP) and underground (UgP) parts. The phytochemical constituents of various *P. autumnale* extracts were analyzed using LC-MS/MS, and their phenolic content was quantified. The biological activities were evaluated through in vitro assays—including antioxidant, anti-inflammatory, acetylcholinesterase-inhibitory, and photoprotection assessments—and in vivo experiments, including evaluations of acute oral toxicity, anti-inflammatory, and analgesic effects. UgP extracts demonstrated significant antioxidant activity, with the methanolic extract exhibiting the highest reducing and superoxide scavenging capacities. Dichloromethane and ethyl acetate extracts performed exceptionally well in ABTS and DPPH assays. The aqueous extract from AgP exhibited noteworthy anti-inflammatory and analgesic effects, surpassing diclofenac in vitro and demonstrating efficacy in vivo. It also showed considerable acetylcholinesterase inhibition, while the ethyl acetate extract displayed high photoprotective potential. The acute toxicity was moderate (LD_50_: 300–400 mg/kg), indicating dose-dependent risks. LC-MS/MS analysis revealed diverse phenolics potentially contributing to both therapeutic and adverse effects. This research enhances the medicinal prospects of *P. autumnale*, provides new perspectives on plant utilization, and suggests its potential as a natural anti-inflammatory agent. However, due to moderate toxicity and dose-dependent effects, cautious application is advised. These findings underscore the importance of toxicological evaluation alongside bioactivity screening in ethnopharmacology to ensure safety.

## 1. Introduction

Medicinal plants represent one of the most relevant sources and bases for the effectiveness of both conventional and modern medicine. According to a recent World Health Organization (WHO) report, around 80% of people worldwide use phytotherapy for their primary healthcare needs to manage or treat medical disorders [1]. The main advantages of medicinal plants appear to be inexpensiveness, minimal risk of serious side effects, and perceived effectiveness [2]. The rich plant kingdom can serve as a new source of multiple chemicals with important biological activity. More recently, comprehensive research on various plant species and their medicinal attributes has led to a global re-evaluation of traditional medicine [3]. Only 15% of the plant species cultivated globally have been studied for their potential medical use, although about 70,000 plant species are known to be used to treat illnesses. Given this low percentage, 25% of the traditional medications applied in contemporary medicine have botanical origins [4]. Under these circumstances, it may imply that further research into naturally occurring sources with potential medical applications remains necessary.

One of the most significant medical discoveries and advancements of the last 20 years has been the realization that inflammatory processes and the immune system are implicated in a broad range of physical and mental health problems that account for the majority of global morbidity and death currently [5]. The immune system uses inflammation as a defense against dangerous stimuli through the naturally occurring innate immune system, which can be divided into three steps: acute inflammation, subacute inflammation, and chronic inflammation [6,7]. Chronic inflammatory diseases are now recognized as the primary cause of death globally, responsible for more than 50% of all fatalities. These include ischemic heart disease, stroke, cancer, diabetes mellitus, chronic kidney disease, non-alcoholic fatty liver disease, autoimmune, and neurodegenerative diseases (such as Alzheimer’s and Parkinson’s) [8]. Given these statistics, research should optimize or discover new sources of drugs to reduce the causes of these diseases. Free radicals are another factor that induces inflammation within humans via cellular damage. On the other hand, Chronic inflammation produces many free radicals that ultimately increase inflammation; this incessant cycle could damage various systems within the human body [9]. Comprehensive research demonstrates that phenolics, a primary class of natural compounds, possess anti-inflammatory capabilities. Plant-derived phenolics, particularly flavonoids and homoisoflavonoids, might reduce acute or chronic inflammation by decreasing oxidative stress and pro-inflammatory conditions [10]. The ability of natural flavonoids to act as both direct and indirect antioxidants, complementing their roles as anti-inflammatory and immunomodulatory agents, provides the potential for photoprotection due to their UV-absorbing characteristics, suggesting exciting prospects for the advancement of photoprotection [11].

**Figure 1:**
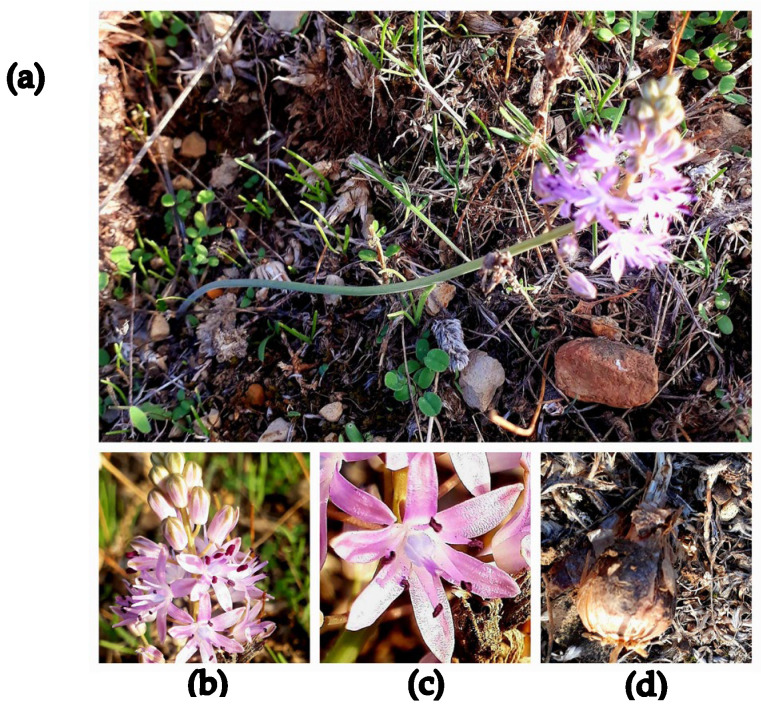
Different parts of *Prospero autumnale* L. were used in this investigation. (**a**) Aerial ground part, AgP; (**b**,**c**) flower; and (**d**) underground part, UgP [pictures taken by Pr. Belguidoum Mahdi in September 2021, Setif, Algeria].

Research utilizing ethnopharmacology represents one of the most efficacious methodologies for the development of novel pharmaceuticals. Approximately 485 plant species across 100 families are traditionally employed as medicinal agents to treat inflammation-related conditions. The *Asparagaceae* family contains the *Scilla* genus as part of these plants [12]. The traditional African applications of *Asparagaceae* plants underscore their effectiveness in managing inflammatory diseases. Plants classified within the genus *Scilla* have also been employed in traditional folk medicine to enhance blood circulation, address neurological conditions, treat infertility in women, and decrease inflammation [13]. Traditional South African applications of *Scilla natalensis* include therapies for gastrointestinal ailments, sprains and fractures, malignant tumors, menstrual pain, and infertility [14]. Conversely, *Scilla nervosa* is utilized for female infertility, constipation, dysentery, nervous disorders, rheumatic fever, and pain [15]. In northeastern Algeria, specifically in Djebel Zdimm (Setif), the endemic species is *Scilla lingulata* Poiret, which is used for menopause and gynecological issues. Nonetheless, comprehensive research on the molecular, morphological, and karyological features has indicated that the genus *Scilla* should be subdivided into distinct new genera, one of which is the genus *Prospero*, which is distributed mostly through southern England and the Mediterranean basin to the Caucasus and northern Iraq [16]. This genus has not previously undergone testing regarding the traditional applications associated with *Silla* or their potential toxicity. In the current study, *Prospero autumnale* L. (Figure 1) is used as a case study to validate these traditional applications safely and explore its potential as a novel tool for drug discovery. *Scilla* plants are characterized by homoisoflavonoids as a primary phenolic compound, which significantly influence their antioxidant, anti-inflammatory, acetylcholinesterase inhibitory, and dermatological properties. Extensive research has demonstrated that homoisoflavonoids inhibit nitric oxide (NO) production in macrophage cells, thereby reducing pro-inflammatory mediators such as NO, iNOS, COX-2, and cytokines (TNF-α, IL-6). Furthermore, these compounds are known to inhibit tyrosinase activity and impede melanosome transfer, rendering them effective in the treatment of hyperpigmentation [17]. A study conducted by Kırmızıbekmez et al. (2025) highlights the presence of both known and novel homoisoflavonoids and stilbenes in *P. autumnale* [18]. Collectively, these findings suggest that the plant possesses significant therapeutic potential, which will be further explored and validated in this investigation.

## 2. Results

### 2.1. Phytochemical Content Analysis

#### 2.1.1. LC-MS/MS

**Figure 2:**
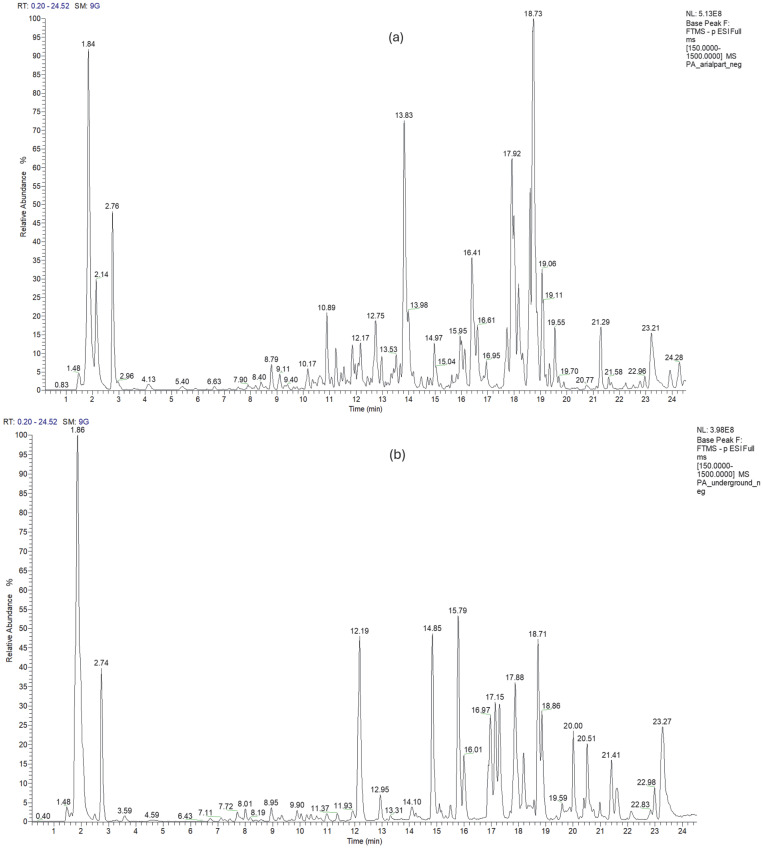
Base peak LC-MS chromatogram profiles of HMEx of *P. autumnale* AgP (**a**) and UgP (**b**) parts analyzed by LC-ESI (−)-HR-MS/MS (LC-Qexactive) in negative ion mode. Base peak chromatograms were generated using Xcalibur software. The *y*-axis represents the relative abundance, expressed as the percentage of the most intense signal (base peak intensity normalized to 100%) within the chromatogram.

**Figure 3:**
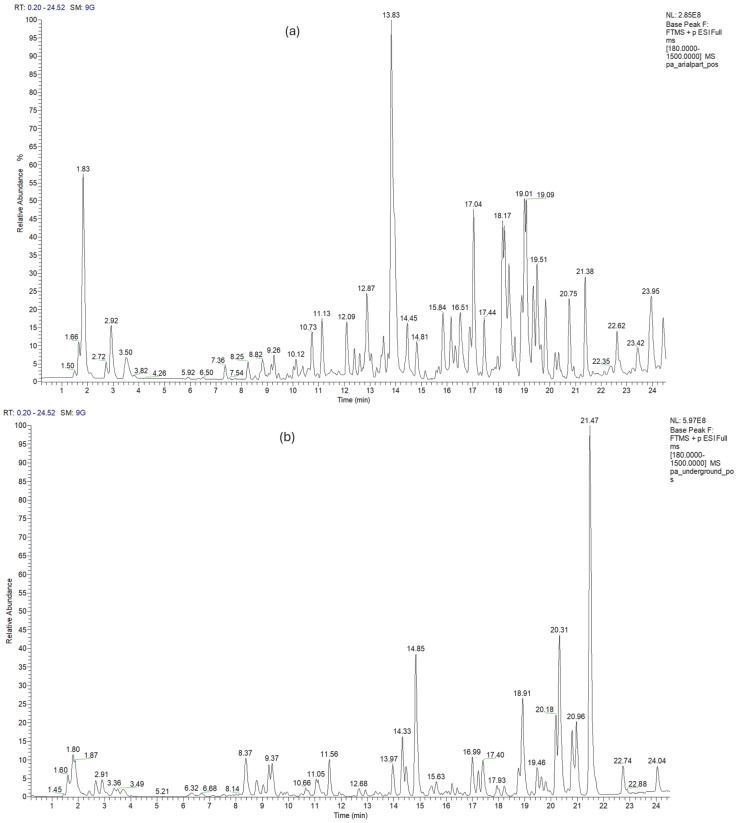
Base peak LC-MS chromatogram profiles of HMEx of *P. autumnale* AgP (**a**) and UgP (**b**) parts analyzed by LC-ESI (+)-HR-MS/MS (LC-Qexactive) in positive ion mode. The *y*-axis represents the relative abundance, expressed as the percentage of the most intense signal (base peak intensity normalized to 100%) within the chromatogram.

The LC-MS profiles in both negative and positive ion modes, as shown in Figure 2 and Figure 3, exhibited multiple peaks. By leveraging accurate mass, molecular formulas, MS/MS fragmentation data, and database searches—together with the literature references—a total of 35 metabolites were tentatively identified. These metabolites were detected in the hydro-methanolic extracts (AHMEx and UHMEx) of both the aerial ground part (AgP) and underground part (UgP), as detailed in Table 1, along with their elution times.

#### 2.1.2. Total Phenolic Content Estimation

The total phenolic content (TPC) of the AgP and UgP extracts was expressed as gallic acid equivalent in µg/mg according to the linear equation of a standard curve (y = 0.0034x + 0.1044, R^2^ = 0.9972). The TPC of different extracts in *P. autumnale* ranged from 1.352 to 650.274 µg GAE/mg ex. Table 2 shows that the greatest TPC in AgP and UgP was found in dichloromethane extract (DcEx), with 413.215 ± 15.636 µg GAE/mg ex and 650.274 ± 14.899 µg GAE/mg ex, respectively. However, the UgP had a higher phenolic content than the AgP in all the extracts. Among UgP extracts, the decreasing order was as follows: UDcEx (650.274 ± 14.899 µg GAE/mg ex), ethyl acetate extract (UEaEx) (571.254 ± 9.622 µg GAE/mg ex), methanolic extract (UMEx) (519.392 ± 21.503 µg GAE/mg ex), butanol extract UBEx (357.725 ± 36.476 µg GAE/mg ex), aqueous phase extract (UAPh) (101.941 ± 6.739 µg GAE/mg ex), aqueous extract (UAEx) (5.47 ± 0.588 µg GAE/mg ex), and petroleum ether extract (UPeEx) (1.352 ± 1.282 µg GAE/mg ex). Meanwhile, for AgP extracts, the decreasing order was as follows: ADcEx (413.215 ± 15.636 µg GAE/mg ex), ABEx (167.137 ± 17.554 µg GAE/mg ex), AEaEx (159.49 ± 20.211 µg GAE/mg ex), AAPh (95.862 ± 6.868 µg GAE/mg ex), AAEx (66.941 ± 1.018 µg GAE/mg ex), and APeEx (10.274 ± 2.668 µg GAE/mg ex).

#### 2.1.3. Total Flavonoid Content Estimation

The flavonoid content was estimated as a quercetin equivalent using the following equation: y = 0.0048x, with R^2^ = 0.997. Furthermore, all results are presented in Table 2.

AgP’s TFC showed the same increasing order as the TPC; specifically, ADcEx exhibited a high TFC (231.94 ± 3.907), while APeEx had a low TFC (13.263 ± 5.745). Conversely, in UgP, UAEx (19.930 ± 2.651) and UMEx (13.263 ± 5.745) had the lowest and highest TFC, respectively. TFC in UgP differed from TPC in the following decreasing order: UMEx (314.652 ± 20.771), UEaEx (295.138 ± 3.240), UDcEx (263.402 ± 47.287), UBEx (102.430 ± 7.954), UAPh (48.958 ± 3.977), UPeEx (38.680 ± 5.008), and UAEx (19.930 ± 2.651).

The statistical analysis showed highly significant effects of extract type (F = 1069.930, *p* < 0.001), plant part (F = 691.704, *p* < 0.001), and their interaction (F = 225.165, *p* < 0.001) on total polyphenol content (TPC). These results demonstrate that TPC differed notably among extract types and plant parts, with the effect of extract type depending on the specific plant part. Similarly, the analysis for total flavonoid content (TFC) revealed highly significant effects of extract type (F = 146.579, *p* < 0.001), plant part (F = 13.084, *p* = 0.001), and their interaction (F = 25.047, *p* < 0.001). This indicates that TFC varied significantly across extracts and plant parts, and the impact of extract type was influenced by the plant part considered.

### 2.2. In Vitro Activities

#### 2.2.1. Antioxidant Activities

**Figure 4:**
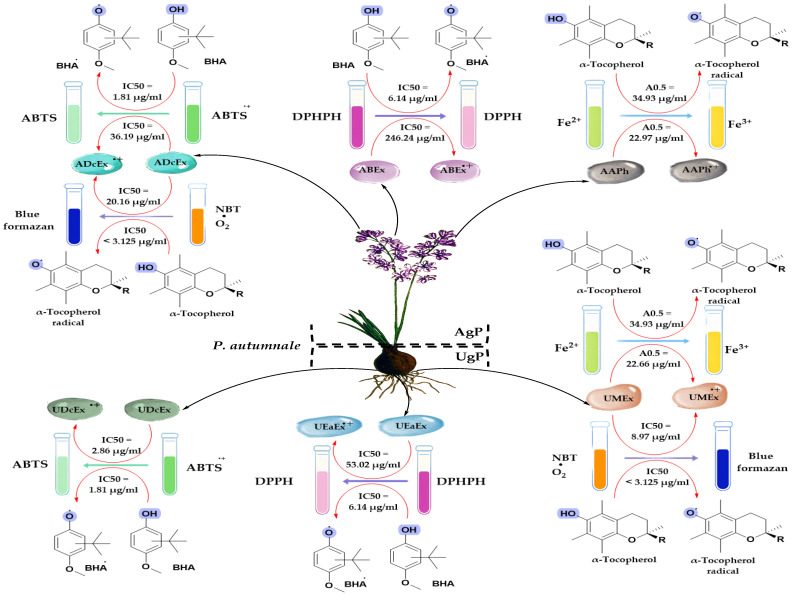
The most potent antioxidant extracts in AgP and UgP of *P. autumnale* across the four assays in comparison with the standards.

The antioxidant properties of *P. autumnale* extracts were assessed using methods that measured their ability to scavenge free radicals (DPPH^•^, ABTS^•+^, and O_2_^•−^-) and to chelate transition-metal ions (reducing power assay). Combining these approaches provided a more comprehensive understanding of the antioxidant capacity of the tested extracts from both AgP and UgP plant parts. Results are presented in Table 3 as IC_50_ and A_0.5_. Additionally, Figure 4 shows the extracts exhibiting the most potent antioxidant activity in both parts.

##### ABTS Radical Scavenging Assay

The scavenging capacities of several extracts of *P. autumnale* and the standard antioxidants (BHA and BHT) were assessed by measuring the reduction of the ABTS^•+^ radical cation through the decolorization process, which was expressed as the % inhibition of absorbance at 734 nm. Regarding the IC_50_ data shown in Table 3, the increasing order of activity for AgP extracts was as follows: APeEx (IC_50_ = 649.97 ± 15.33 µg/mL), AAEx (IC_50_ = 243.63 ± 3.55 µg/mL), AAph (IC_50_ = 160.16 ± 5.69 µg/mL), AEaEx (IC_50_ = 97.09 ± 2.87 µg/mL), ABEx (IC_50_ = 92.6 ± 3.39 µg/mL), and ADcEx (IC_50_ = 36.19 ± 0.83 µg/mL), while the UgP extracts followed this order: UPeEx (IC_50_ = 4804.37 ± 32.11 µg/mL), UAEx (IC_50_ = 652.93 ± 13.66 µg/mL), UAph (IC_50_ = 252.86 ± 12.84 µg/mL), UBEx (IC_50_ = 72 ± 0.55 µg/mL), UEaEx (IC_50_ = 26.11 ± 0.81 µg/mL), UMEx (IC_50_ = 16.28 ± 0.6 µg/mL), UDcEx (IC_50_ = 2.86 ± 0.71 µg/mL), which were comparable to BHA (IC_50_ = 1.81 ± 0.10 µg/mL) and BHT (IC_50_ = 1.29 ± 0.30 µg/mL), respectively. The results indicated that ADcEx and UDcEx exhibited significantly higher ABTS radical scavenging activity in the two parts of *P. autumnale* (Figure 4).

##### DPPH Radical Scavenging Assay

Another test was performed to evaluate the scavenging activities of AgP and UgP extracts against the free radicals of DPPH. Results (Table 3) showed that the neutralization of the DPPH radical was achieved by ABEx (IC_50_ = 246.24 ± 5.39 µg/mL), followed by AAph (IC_50_ = 346.31 ± 4.44 µg/mL) from AgP, whereas UEaEx (IC_50_ = 53.02 ± 1.01 µg/mL), UDcEx (IC_50_ = 133.92 ± 3.47 µg/mL), and UMEx (IC_50_ = 140.01 ± 4.50 µg/mL) from UgP. These extracts were found to possess strong antioxidant abilities compared to other extracts, which showed lower scavenging effect, depending on the activity with α-tocopherol (IC_50_ = 13.02 ± 5.17 µg/mL) and the commonly used synthetic antioxidants BHT (IC_50_ = 12.99 ± 0.41 µg/mL) and BHA (IC_50_ = 6.14 ± 0.41 µg/mL) (Figure 4).

##### Superoxide Radical Scavenging Activity (NBT)

The alkaline DMSO test is employed to produce superoxide by introducing NaOH to air-saturated DMSO. The produced superoxide anion reduces nitroblue tetrazolium (NBT) to form the blue formazan 0 [19]. The decreased absorbance at 560 nm in the presence of antioxidants indicates that superoxide radicals are consumed in the reaction mixture, preventing the formation of blue NBT. Table 3 displays the relative effectiveness of several extracts from *P. autumnale* and standards in neutralizing superoxide radicals. The activity of AgP followed the order: APeEx (IC_50_ = 104.91 ± 18.55 µg/mL), AAPh (IC_50_ = 63.77 ± 4.92 µg/mL), AEaEx (IC_50_ = 27.20 ± 1.79 µg/mL), ABEx (IC_50_ = 24.03 ± 2.74 µg/mL), AAEx (IC_50_ = 23.03 ± 3.89 µg/mL), ADcEx (IC_50_ = 20.16 ± 3.57 µg/mL). In contrast, the order of activity in UgP was: UAPh (IC_50_ = 89.61 ± 2.33 µg/mL), UAEx (IC_50_ = 78.70 ± 16.38 µg/mL), UPeEx (IC_50_ = 56.55 ± 4.52 µg/mL), UDcEx (IC_50_ = 16.85 ± 0.49 µg/mL), UBEx (IC_50_ = 11.15 ± 0.21 µg/mL), UEaEx (IC_50_ = 9.84 ± 0.60 µg/mL), UMEx (IC_50_ = 8.97 ± 0.78 µg/mL). When compared with the standards, Tanic acid (IC_50_ ˂ 3.125 µg/mL) and α-Tocopherol (IC_50_ ˂ 3.125 µg/mL), the ADcEx (IC_50_ = 20.16 ± 3.57 µg/mL) and UMEx (IC_50_ = 8.97 ± 0.78 µg/mL) from AgP and UgP, respectively, showed the strongest scavenging activity against the O_2_^•−^ radical (Figure 4).

##### Reducing Power (FRAP)

The reducing power of *P. autumnale* extracts was assessed by the conversion of the ferric ion (Fe^3+^)-ligand complex into the intensely blue ferrous (Fe^2+^) complex, which was detected by an increase in absorbance at 593 nm. Table 3 summarizes the reducing activity of AgP extracts through their electron transfer ability, which increased in the following order: ADcEx (A_0.5_ = 346.34 ± 29.25 µg/mL), APeEx (A_0.5_ = 186.60 ± 1.73 µg/mL), ABEx (A_0.5_ = 139.91 ± 2.03 µg/mL), AEaEx (A_0.5_ = 109.34 ± 5.43 µg/mL), AAEx (A_0.5_ = 34.94 ± 2.31 µg/mL), AAPh (A_0.5_ = 22.97 ± 0.20 µg/mL). On the other hand, the SET activity of the UgP extracts was as follows: UPeEx (A_0.5_= 791.39 ± 59.07 µg/mL), UAPh (A_0.5_ = 353.73 ± 44.40 µg/mL), UAEx (A_0.5_ = 212.31 ± 16.91 µg/mL), UEaEx (A_0.5_ = 166.66 ± 2.53 µg/mL), UDcEx (A_0.5_ = 147.60 ± 1.79 µg/mL), UBEx (A_0.5_ = 47.93 ± 8.50 µg/mL), UMEx (A_0.5_ = 22.66 ± 0.53 µg/mL). Overall, both AAPh and UMEx from AgP and UgP, respectively, showed the strongest ability to chelate Fe^2+^ more than α-Tocopherol (A_0.5_ = 34.93 ± 2.38 µg/mL) and lower than Ascorbic acid (A_0.5_ = 6.77 ± 1.15 µg/mL), used as standards (Figure 4).

The notable extract × plant-part interactions observed in ABTS, NBT, and FRAP assays indicate that antioxidant activity was not determined solely by the plant part or extract type independently, but rather by the specific combination of both factors. Overall, UgP extracts demonstrated stronger activity in numerous assays, particularly UDcEx, UMEx, UEaEx, and UBEx. However, this trend was not universal. In ABTS and FRAP assays, AAEx and AAPh exhibited greater activity than their corresponding UgP extracts. These results suggest that antioxidant activity is contingent upon the solvent or extract type employed for each plant part. The pronounced activity of UDcEx in ABTS, alongside the substantial reducing power of UMEx, AAPh, AAEx, and UBEx in FRAP, implies that different extract types may concentrate distinct antioxidant compounds. This may account for the variability in performance of the same plant part across different assays. Given that ABTS, DPPH, NBT, and FRAP assays are based on differing reaction mechanisms, observed differences among them are anticipated and should not be construed as contradictions.

#### 2.2.2. Anti-Inflammatory Activity (Bovine Serum Albumin Denaturation)

**Figure 5:**
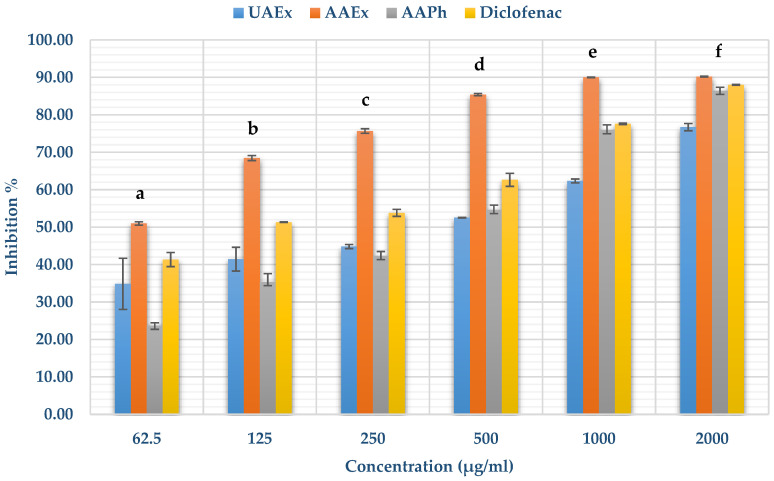
Protein denaturation inhibition of *P. autumnale* extracts and diclofenac standard. Values marked with different superscripts (a–f) are statistically significantly different (*p* < 0.05).

The anti-inflammatory effects of the investigated aqueous extracts of *P. autumnale* were assessed using the in vitro BSA protein denaturation assay, with results summarized in Figure 5 and Table 4. AAEx demonstrated strong inhibition of protein denaturation of 90.20%, 90.00%, 85.39%, 75.66%, 68.45%, and 50.98% at doses of 2000, 1000, 500, 250, 125, and 62.5 μg/mL, respectively, in comparison with diclofenac sodium, a standard, which exhibited 88.01%, 77.58%, 62.63%, 53.82%, 51.33%, and 41.33% at the corresponding doses. Conversely, both UAEx (44.81%) and AAPh (42.32%) displayed moderate inhibition at 250 μg/mL. Additionally, statistical analysis revealed highly significant effects of extract type and concentration on the percentage of inhibition. A significant main effect of extract was identified (F(3, 48) = 730.077, *p* < 0.001, partial η^2^ = 0.979), indicating considerable differences in inhibitory activity among the tested extracts and diclofenac. The concentration also exhibited a highly significant effect (F(5, 48) = 1150.312, *p* < 0.001, partial η^2^ = 0.992), demonstrating a strong dose-dependent response. Furthermore, the interaction between extract type and concentration was significant (F(15, 48) = 32.970, *p* < 0.001, partial η^2^ = 0.912), implying that the extent of the concentration effect varied according to the extract examined. Accordingly, the findings demonstrated that AAEx (IC_50_ μg/mL 58.97 ± 1.71) substantially inhibited thermal protein denaturation compared with the NSAID diclofenac sodium (IC_50_ μg/mL = 109.45 ± 1.24), thereby indicating its notable anti-inflammatory properties.

#### 2.2.3. Acetylcholinesterase Inhibition (Alzheimer’s Disease)

The summary of acetylcholinesterase inhibition by polar and non-polar extracts from the AgP and UgP parts of *P. autumnale* used in the current study is presented in Table 5. The acetylcholinesterase inhibition by each extract was principally expressed by its IC_50_ value. Only AAEx (IC_50_ µg/mL = 11.97 ± 0.62), followed by UMEx (IC_50_ µg/mL = 25.98 ± 0.57) for AgP and UgP, respectively, showed strong inhibition compared to galantamine (IC_50_ µg/mL = 6.27 ± 1.15) as a standard drug. In contrast, no inhibition is observed in APeEx, UPeEx, AAPh, and UAEx.

#### 2.2.4. Photoprotection Activity (Sun Protection Factor)

**Figure 6:**
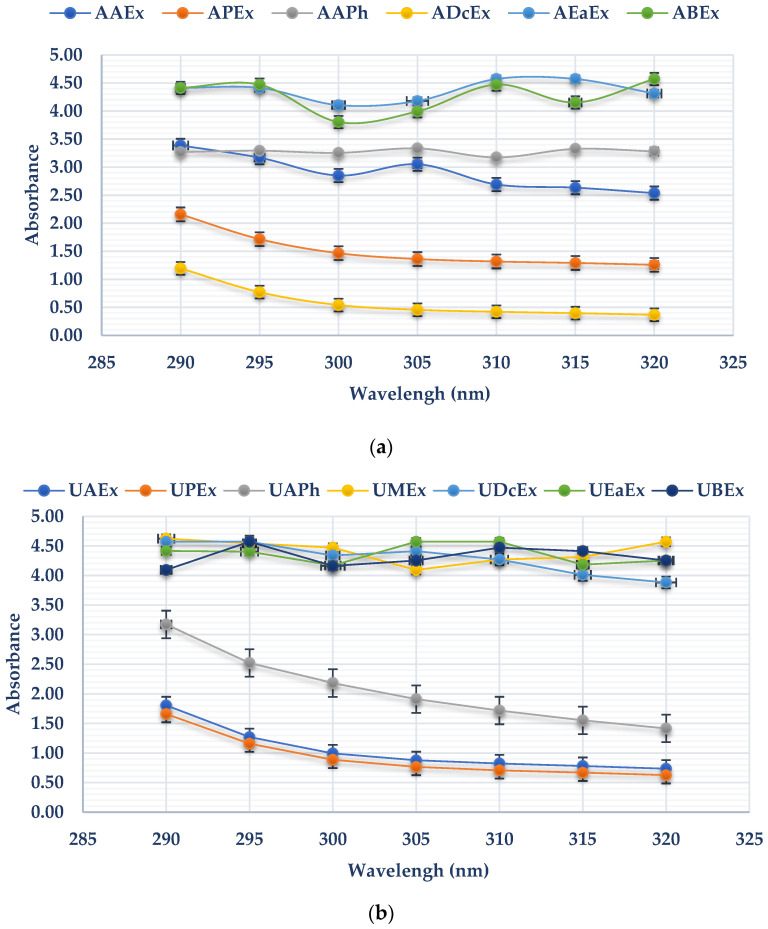
(**a**) Absorbance of AgP extracts, and (**b**) absorbance of UgP extracts, in the (290–320 nm) range.

The photoprotection activity of several extracts from AgP and UgP of *P. autumnale* was evaluated by UV spectrophotometry, applying the Mansur methodology. Each sample was measured to evaluate the SPF value of sunscreens containing a wide variety of chemicals that have specific absorbance in some parts of the UV spectrum. Figure 6 shows the UV absorption of the samples, and Table 6 summarizes their SPF values. Based on Commission Recommendation 2006/647/EC, AAEx, UAEx, APeEx, UPeEx, AAPh, and UAPh from the two parts have low to medium sun protection with decreasing UV absorption. On the other hand, UDcEx, UBEx, AEaEx, UMEx, followed by ABEx, have high UVB protection, while UEaEx (44.01 ± 1.60) has the highest protection, which explains the maximum absorption in some wavelength in the 280–320 nm range.

The statistical analysis demonstrated a significant impact of wavelength on absorbance (F(6, 132) = 19.920, *p* < 0.001), signifying a spectral dependence on wavelength. A notable interaction was observed between wavelength and extract type (F(36, 132) = 3.224, *p* < 0.001), indicating that absorbance profiles varied among the seven extracts. The interaction between wavelength and plant part was not statistically significant (F(6, 132) = 1.404, *p* = 0.218), suggesting that spectral patterns were consistent across different plant parts. Overall, the primary factors influencing absorbance were extract type and wavelength, with complex interactions suggesting distinct spectral profiles for each extract–plant part combination, which may potentially influence their photoprotective and SPF properties. The analysis also revealed a significant effect of extract type on SPF (F(6, 23) = 613.901, *p* < 0.001), indicating variability in SPF among the extracts. The main effect of plant part was not significant (F(1, 23) = 1.538, *p* = 0.227), implying that AgP and UgP did not differ significantly when considered independently. However, the interaction between extract type and plant part was highly significant (F(5, 23) = 429.219, *p* < 0.001), meaning that the influence of extract type on SPF was dependent on the plant part, with different extracts exhibiting varied performance from AgP or UgP.

### 2.3. In Vivo Activities

#### 2.3.1. Oral Acute Toxicity

**Figure 7:**
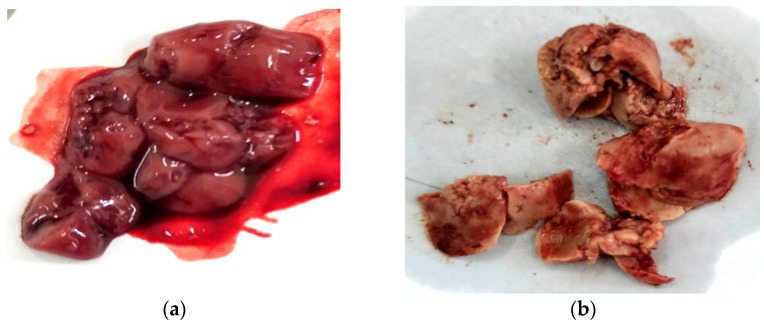
Representative liver autopsy from mice treated in the same conditions with aqueous extracts AAEx and UAEx, obtained from the AgP and UgP parts of *P. autumnale*, respectively: (**a**) mice treated with AAEx at a dose of LD_50_: 400 mg/kg; (**b**) mice treated with UAEx at a dose of LD_50_: 300 mg/kg.

Table 7 summarizes the effects of *P. autumnale* aqueous extract in mice following acute oral administration. Signs of toxicity, including changes in heartbeat and subsequent mortality in mice, were observed when the dosage increased from 200 mg/kg to 500 mg/kg in both extracts. All mice administered UAEx and AAEx at a dosage of 500 mg/kg died within 24 h of administration. Fifty percent mortality was observed at dosages of 300 mg/kg and 400 mg/kg for UAEx and AAEx, respectively. Accordingly, the LD_50_ values of UAEx and AAEx extracts were estimated at 300 and 400 mg/kg, respectively. The effect of the extracts on the relative weights of major organs (Table 7) in relation to body weight (Table 8) revealed a slight increase in body weight at the LD_50_ doses on the 14th day compared to the 1st day. The findings indicated that the liver exhibited adverse effects during the treatment, as evidenced by an increase in weight compared to the control. However, the heart and kidneys maintained normal weight gain without significant variation. The autopsy revealed that the extracts exerted a hepatotoxic effect, with only the liver affected, as shown in Figure 7. At a dosage of 300 mg/kg (Figure 7b), the UAEx extract elicited hepatic ischemia, characterized by the vasoconstriction of hepatic vessels and a consequent reduction in blood flow. This resulted in the liver appearing pale and discolored due to diminished blood content. Conversely, at a dosage of 400 mg/kg (Figure 7a), the AAEx extract induced hepatic congestion accompanied by hemorrhagic phenomena, indicative of toxicity and compromise of hepatic vascular integrity. This was associated with hepatocellular damage, capillary rupture, and increased vascular fragility. As a result, blood leakage occurred, imparting a dark, moist appearance to the liver.

#### 2.3.2. Acute Inflammation (Carrageenan-Induced Paw Edema)

**Figure 8:**
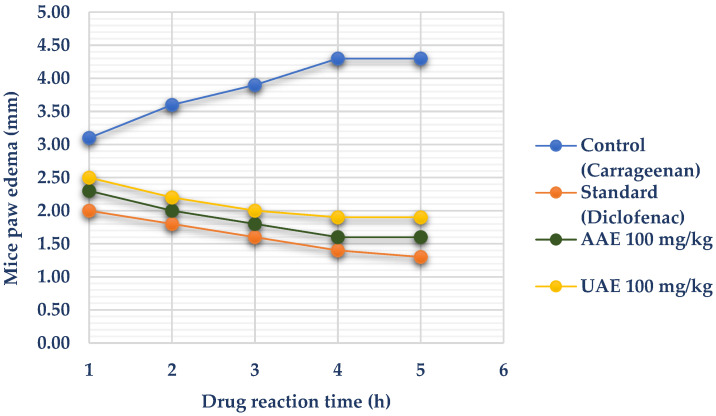
Descriptive kinetic profile of paw edema evolution following treatment with *P. autumnale* extracts.

All rats administered carrageenan exhibited a localized paw edema response. In control mice, the subplantar administration of carrageenan induced localized edema that progressively intensified, reaching a maximum at 3 h post-injection (Figure 8). The oral treatment with both UAEx and AAEx of *P. autumnale* resulted in a significant reduction in paw edema compared to the vehicle-treated control group (Figure 8). The temporal progression of the therapeutic effects of the extracts was analogous to that of diclofenac. The distinctions in clinical anti-inflammatory effectiveness among the experimental groups have been expressed as the percentage suppression of rat paw edema (Table 9). The results indicated that diclofenac, UAEx, and AAEx extracts exhibit equivalent paw edema inhibition capacities. However, AAEx demonstrated superior anti-inflammatory potency compared to UAEx and diclofenac (Table 7).

#### 2.3.3. Analgesic Activity

The intraperitoneal administration of acetic acid resulted in an average of 77 writhes in the control group, 30 min post-injection, allowing the calculation of a pain inhibition percentage in mice that received an oral therapeutic dose of 100 mg/kg of UAEx, AAEx, and paracetamol. The analgesic efficacy of the AAEx extract overtook that of the UAEx extract by demonstrating the reduction in muscle contraction reflexes with inhibitory percentages of 64.63% and 59.98%, respectively, which is similar to the reference drug (paracetamol 64.75%), all results summarized in Table 10.

## 3. Discussion

The phenolic compound family is one of the major groups of substances that serve as principal antioxidants or free radical terminators [20]. Analyzing these metabolites both qualitatively and quantitatively will aid in discovering new, effective plant-based drugs and offer scientific validation for traditional folk remedies [21].

To date, there have been no reports on the application of a liquid chromatography platform coupled to a high-resolution mass spectrometer to evaluate the phytochemical content of HMEx obtained from both AgP and UgP of *P. autumnale.* Only recently, a study by Kırmızıbekmez et al. (2025) conducted an isolation study on the ethanol extract of *P. autumnale* bulbs, which led to the discovery of four novel phenolic compounds, including a stilbene, a homoisoflavonoid derivative, a dimer, and an unprecedented heterodimer, along with six known analogs characterized by NMR spectroscopy and theoretical ECD calculations [18].

In the present study, LC-MS profiles in negative and positive ion modes, reported in Figure 1 and Figure 2, respectively, revealed several peaks. Thanks to accurate mass, molecular formula, MS/MS fragmentation, and research in databases such as Compound Discoverer and the literature, a total of 35 metabolites were putatively identified, most of them in *P. autumnale* extracts, for the first time, distributed across both AgP and UgP, as reported in Table 1 according to their time of elution.

The primary metabolites identified include amino acids such as arginine, phenylalanine, and tryptophan, as well as sugars such as sucrose. Moreover, most of the secondary metabolites identified belong to the class of phenolic compounds, particularly flavonoids (e.g., kaempferol di-glucoside, quercetin acetyl glucoside, and luteolin) and their homoisoflavonoid derivatives, which three of them already reported in UgP of *P. autumnale* by Kırmızıbekmez et al. (2025), namely, (5,7-dihydroxy-3-(4-hydroxybenzyl)chroman-4-one, 5,7-dihydroxy-3-(4-hydroxybenzyl)-8-methoxychroman-4-one, 5,7-dihydroxy-3-(4-hydroxybenzylidene)chroman-4-one (4′-demethyleucomine)) [18]. Other important constituents include phenolic acids, such as ferulic acid, and triterpenoid saponins.

Several metabolites, such as glehlinoside C, vicenin 2, and muscomin, were ubiquitously present in both the AgP and UgP, suggesting a systemic distribution and a potential general role in the plant’s metabolism or defense. In contrast, other metabolites exhibited a marked tissue-specific distribution. For instance, kaempferol diglycoside was exclusive to the aerial tissue AgP, while a homoisoflavonoid was exclusive to the UgP.

Notably, compounds **29** and **34**, due to their chemical structures and fragmentation, were identified as triterpenoid glycosides with high molecular weights (C_59_H_93_O_28_ and C_59_H_94_O_27_, respectively). They were present in high concentrations in the aerial part. Their structures could not be defined in detail by mass spectrometry; accordingly, it is necessary to conduct further purification of the extracts in the future.

Phenolic components have different degrees of solubility in different solvents. In terms of solubility, these compounds may be polar, nonpolar, or mid-polar. For this reason, gradient solvents are essential for classification.

According to the results in Table 2, the TPC and TFC in AgP of *P. autumnale* were abundant in different extracts. It was observed that the effect of solvents on TFC is similar to that on TPC, with ADcEx demonstrating the highest extract content. In comparison to previous studies, Mammadov et al. (2017) found that the TPC and TFC of AgP ethanol extracts were 16.03 ± 0.05 mg GAEs/g and 25.01 ± 0.08 mg GAEs/g, respectively [13]. However, in the older genus, *Scilla*, for example, in the leaves of *Scilla hyacinthina*, the TPC of the methanolic extract was 10.33 ± 0.53 mg GAE/g, while the aqueous extract was 4.48 ± 0.36 mg GAE/g. The TFC of the methanolic extract was 6.23 ± 0.58 mg GAE/g extract, and that of the aqueous extract was 9.32 ± 0.57 mg GAE/g [22].

The TPC order of UgP extracts differed from TFC. This variation may be due to a higher presence of nonphenolic compounds, such as carbohydrates and terpenes, or because of the total flavonoid content, which includes various types of flavonoids, most of which do not chelate with AlCl_3_, making them undetectable by spectrophotometry [23].

In general, the UgP of *P. autumnale* exhibited the highest phenolic and flavonoid contents, surpassing those of AgP, with UMEx and UDcEx showing significant richness. Furthermore, other studies indicate that both UEaEx and UMEx extracts of *P. autumnale* contained greater TPC than *Scilla mesopotamica*, with values of 62.24 µg GAE/mg and 35.89 µg GAE/mg, respectively [24]. Additionally, UgP also yielded the highest TPC compared to *Scilla hyacinthina*, which measured 3.43 µg GAE/mg [25].

This comprehensive phytochemical profiling provides a solid basis for rationalizing the biological activity of *P. autumnale* extracts.

The future prospects of plants as a source of novel pharmaceuticals are demonstrated by their significance in scientific studies for many therapeutic uses [26]. *P. autumnale*, like other medicinal plants, contains several bioactive compounds that may lead to unforeseen or adverse effects, affecting the physiology of various organs. Toxicity refers to the extent of harm resulting from the interaction between cells and a poisonous substance. This relationship fluctuates based on the cell membrane and chemical characteristics of the toxicants, which might affect the extracellular matrix, cell surface, and the underlying tissues [27]. The harmful effects may occur before the toxicants attach to essential organs such as the liver and kidneys. To ensure the safe utilization of *P. autumnale* as a plant-based medication, it is essential to conduct a toxicity assessment in addition to reviewing historical material. There are no ethnobotanical studies demonstrating the toxicity of *P. autumnale*; however, several reports indicate toxic effects in some *Asparagaceae* species involving common symptoms of poisoning encompassing diarrhea, stomach discomfort, emphysema, and an elevated heart rate, which can potentially lead to fatality [28]. According to OECD Guideline 423, the findings of this investigation demonstrated that the aqueous extracts of *P. autumnale* were well-tolerated in mice under an oral dosage of 200 mg/kg. Mortality and other symptoms related to the single oral administration of UAEx and AAEx were observed in mice with dosages starting at 200 mg/kg. The oral acute toxicity test for UAEx and AAEx yielded LD_50_ values of 300 and 400 mg/kg b.w., respectively. Loomis and Hayes’ classification (1996) identifies a chemical substance having an LD_50_ within the range of 50–500 mg/kg as moderately toxic [29]. Under these circumstances, the calculated LD_50_ of *P. autumnale* for the two parts indicates that the plant should be classified as moderately toxic upon acute intake.

The harmful effects may occur before the toxicants attach to essential organs, such as the liver, heart, and kidneys [30], which are analyzed by comparing weight variations from the initial to the last day with those of the control group. The study indicates that the toxic doses of UAEx and AAEx have hazardous effects on the liver, with an increase in weight, which may be due to inflammation. The complicated chemical composition of medicinal plants may lead to moderate to significant side effects from herbal medicine usage. The five main groups of toxic compounds are alkaloids, followed by glucosides, terpenoids, peptides, and phenolics [26]. Phytochemical investigations of *P. autumnale* extracts reveal a high richness of triterpenoids and phenolic compounds such as phenolic acids, flavonoids, homoisoflavonoids, and stilbenes, which may either separately or together cause toxic effects.

The excessive reactive oxygen and nitrogen species (ROS/RNS) generated under abnormal conditions cause oxidative stress. This stress can result in cellular damage and represent a risk to biological systems. The role of antioxidants in daily life is essential since they may scavenge free radicals, events induced by oxidative stress [31]. Therefore, the significant results in TPC and TFC from various extracts in *P. autumnale* have encouraged us to study their antioxidant activities as a novel source of them. The biological activity of polyphenols depends on their solubility, metabolism, absorption, structures, combination with other compounds in complex systems, conformational structures, and their mechanisms to inhibit free radicals [32]. Consequently, variable experimental conditions with different principles were used to quantify different oxidation products. Exceptions to the reducing power test, ABTS, DPPH, and DMSO alkaline tests were reported for the first time in the current study.

The DPPH assay is the most common and widely used method to scavenge oxidants. The current findings indicate that only ABEx from AgP (IC_50_ µg/mL = 246.24 ± 5.39) and UEaEx (IC_50_ µg/mL = 53.02 ± 1.01) from UgP demonstrated significant scavenging activity compared to the other extracts. The acceptable antioxidant activity of these extracts may be attributed to the high levels of lipophilic compounds and their ability to act as HAT/SET. In contrast, other species of the *Scilla* genus exhibited strong inhibition against the DPPH radical. A study by Alluri et al. (2015) shows that methanolic extracts from different parts of *Scilla hyacinthina* (IC_50_ µg/mL = 19.76 ± 0.89 for leaves, 22.73 ± 0.73 for bulbs) were more effective in scavenging than aqueous extracts (IC_50_ µg/mL = 23.75 ± 0.52 for leaves, 25.44 ± 0.68 for bulbs) [22]. On the other hand, Özen et al. (2022) found that the ethyl acetate extract of leaves and bulbs from *Scilla bifolia* contained 9.17 μmol Trolox/g and 2.59 μmol Trolox/g, respectively [33].

Extracts that demonstrate insufficient antioxidant activity using one method should not be classified as ineffective sources of antioxidants prior to further testing with another method. Consequently, based on the ability of ABTS to determine both hydrophilic and lipophilic compounds across a wide range of pH levels [34], most of the *P. autumnale* extracts exhibit strong scavenging activity. The DcEx of both AgP (IC_50_ = 36.19 ± 0.83 µg/mL) and UgP (IC_50_ = 2.86 ± 0.71 µg/mL) act as fast and effective scavengers of the ABTS radical through HAT/SET, in comparison with the standards BHT (IC_50_ = 1.29 ± 0.30 µg/mL) and BHA (IC_50_ = 1.81 ± 0.10 µg/mL). The report by Alluri et al. (2015) on *Scilla hyacinthina* indicated that the methanolic extract (IC_50_ µg/mL = 21.02 ± 0.72 leaves, 23.48 ± 0.63 bulbs) was less effective as a scavenger than UMEx (IC_50_ µg/mL = 16.28 ± 0.6, UgP) of *P. autumnale*, whereas the aqueous extracts (IC_50_ µg/mL = 24.56 ± 0.81 leaves, 25.96 ± 0.79 bulbs) exhibited greater scavenging activity than both parts of *P. autumnale* [22].

DPPH and ABTS are synthetic radicals with structures that differ from those of the reactive oxygen species present in aerobic biological systems. For this reason, the DMSO alkaline method is useful because it assesses the ability of antioxidants found in *P. autumnale* extracts to remove superoxide radicals under physiological conditions. Superoxide anion is essential in the generation of several reactive oxygen species, including the hydroxyl radical (HO^•^), hydrogen peroxide (H_2_O_2_), and singlet oxygen (O_2_^•−^), which cause oxidative damage to DNA, proteins, and lipids [35]. Several extracts of *P. autumnale* parts possessed significantly higher superoxide radical scavenging. UMEx (IC_50_ µg/mL = 8.97 ± 0.78) and ADcEx (IC_50_ µg/mL = 20.16 ± 3.57) had the highest level of radical scavenging; however, antioxidants present in most of the UgP extracts had stronger inhibition than those present in the AgP extracts, which may be due to their being good electron donors.

**Figure 9:**
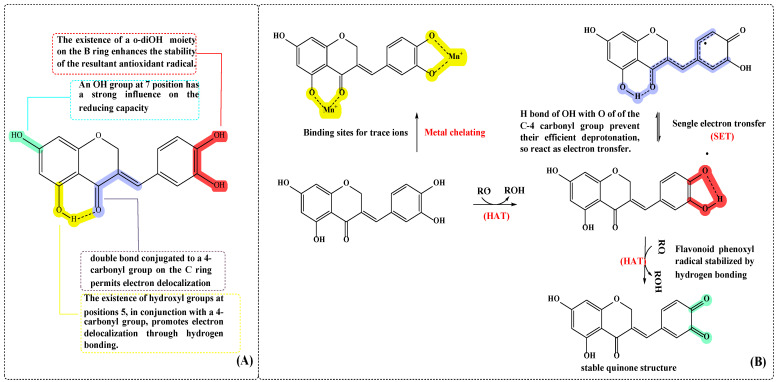
Proposal of antioxidant activity of (E)-3-(3,4-dihydroxybenzylidene)-5,7-dihydroxychroman-4-one; (**A**) key factors of antioxidant activity; and (**B**) mechanism of reaction by SET, HAT, and metal chelation, based on the structure–activity relationship of the flavonoid.

According to LC-MS/MS results, both UHMEx and AHMEx of *P. autumnale* are rich in phenolic compounds such as flavonoids, which have strong antioxidant activities closely related to their skeletons. Based on structure–activity relationship SAR studies, the structural prerequisites for HAT comprise an ortho-dihydroxy substitution in the B ring, which confers higher stability to the radical form, and a C2-C3 double bond in conjunction with a carbonyl group at C-4 in the C ring, which is responsible for electron delocalization [23]. These backbone structure characters are found on detected flavonoids such as luteolin, rhamnetin 3-glucoside, 6-methoxyluteolin 7-glucoside, quercetin 3-methyl ether, and their derivative homoisoflavonoids (*E*)-3-(3,4-dihydroxybenzylidene)-5,7-dihydroxychroman-4-one (Figure 9) and (*E*)-3-(3,4-dihydroxybenzylidene)-5,7-dihydroxy-6-methoxychroman-4-one, 5,7-dihydroxy-3-(4-hydroxybenzylidene)chroman-4-one (4′-demethyleucomine). The extensive variation in the number, type, and position of substituents allows the molecule to participate in several reaction forms. Luteolin, vicenin 2, apigenin, dihydrokaempferol, and the seven homoisoflavonoid derivatives have the ability to act as SET, which could be caused by the strong hydrogen bond between the 3/5-OH group and the oxygen atom of the C-4 carbonyl group, which prevents the H-donation [23]. The electron donation method may also apply to monohydroxyflavones, such as cerarvensin, dihydrokaempferol, genistein, and apigenin, when hydrogen atoms cannot be donated from other hydroxyl groups. These SAR studies may demonstrate the extract’s strongest antioxidant activity.

The importance of chelating Fe^2+^ comes from its ability to strongly accelerate lipid peroxidation through rapidly reacting with hydrogen peroxide (H_2_O_2_) to produce very harmful hydroxyl radicals or react with oxygen to produce O_2_^•−^ by the Haber–Weiss reaction [35,36]. Results observed in reducing power assay suggested that the ability to chelate iron metal by *P. autumnale* is present in the strong reducing antioxidant content of UMEx (IC_50_ µg/mL = 22.66 ± 0.53) and AAPh (IC_50_ µg/mL = 22.97 ± 0.20). Other results by Mammadov et al. (2017) reported that the ethanol extract of *P. autumnale* from UgP and AgP were IC_50_ = 5.03 ± 0.07 mg EDTAEs/g and IC_50_ = 7.14 ± 0.11 mg EDTAEs/g, respectively [13]. Both UgP and AgP contain several flavonoids and homoisoflavonoids, which have the ability to contribute Fe ^2+^ chelation in the 3′,4′-diOH group in the B ring, in addition to 3/5-OH groups and the 4-carbonyl group (Figure 9) [37], such as rhamnetin 3-glucoside, kaempferol, 6-Methoxyluteolin 7-glucoside, and the two homoisoflavonoids (compounds **26** and **28**).

The report by Tian et al. (2021) exhibits that the IC50 DPPH values of kaempferol, luteolin, quercetin, VC and BHT were 5.318, 2.099, 1.84, 3.028, and 10.5 µg/mL, respectively, whereas the IC_50_ ABTS values of kaempferol, luteolin, apigenin, quercetin, VC, and BHT were 0.8506, 0.59, 0.8243, 0.5083, 2.1563, and 1.4497 µg/mL, respectively; meanwhile, the FRAP values for the six compounds ranged from 0.0101 to 0.0402 mmol Fe^2+^/µg/mL [38]. Studies on homoisoflavonoids indicate that the benzylidene-4-chromanones type had strong antioxidant activity, more than commercial antioxidants BHA, BHT, VE, and VC, in superoxide and DPPH free radical scavenging methods [39,40]. Other studies confirm the strong antioxidant activity of benzylidene-4-chromanones by measuring their inhibitory effect on enzymatically induced lipid peroxidation in vitro by Fe^2+^/Ascorbic acid, which was stronger than the BHT and stobadiene standards [41]. Phenolic acids have also been effective antioxidants, finding that Ferulic acid (10 mg/kg) not only scavenges free radicals but also improves the activity of enzymes that are responsible for scavenging free radicals, and it inhibits enzymes that catalyze the formation of free radicals [42].

Inflammation is a complex biological process that occurs when vascular tissues are harmed by damaging stimuli, such as physical trauma, toxic chemicals, or microbial agents. This response is initiated by the release of chemical mediators from damaged tissue and migratory cells [3]. This response requires modifications in blood flow, and it increases the permeability of vascular tissues and protein denaturation, membrane modification, and tissue degradation through the activation and movement of leucocytes with the production of reactive oxygen species (ROS), as well as local inflammatory agents such as prostaglandins, leukotrienes, and platelet-activating factors induced by phospholipase A2, COX, and LOX towards the site of injury [43]. These factors induce symptoms such as fever, redness, swelling, and pain, as well as disruptions in normal physiological functions [3]. Chronic inflammation can result in the development of rheumatoid arthritis, atherosclerosis, hay fever, and ischemic heart disorders [44].

Non-steroidal anti-inflammatory drugs (NSAIDs) such as indomethacin, ibuprofen, flufenamic acid, and salicylic acid are widely used to treat pain and reduce swelling caused by inflammation [45]. These analgesic drugs frequently offer only a 50% reduction in pain for around 30% of individuals, who react by inhibiting the formation of endogenous prostaglandins by the blockage of the COX enzyme, as well as by preventing protein denaturation [12,46]. Nevertheless, these drugs can produce unfavorable side effects in the cardiovascular system, gastrointestinal tract, and renal function [12]. Given the circumstances, it is important to develop novel drugs that can effectively address inflammation while minimizing possible side effects.

The carrageenan-induced inflammation model is frequently used to evaluate new anti-inflammatory drugs. The injection of carrageenan into the subplantar surface of the mice paw elicited a biphasic edema; the initial phase is marked by the release of histamine, serotonin, and kinins within the first hour, whereas the subsequent phase is associated with the release of prostaglandins and several cytokines, including IL-1β, IL-6, IL-10, and TNF-α, between 2 and 4 h [46,47]. The second phase is responsive to many clinically effective anti-inflammatory drugs (NSAIDs), which mainly block cyclooxygenase, implicated in prostaglandin formation [46]. The findings of the current investigation indicate that AAEx and UAEx at the dose of 100 mg/kg significantly reduced paw size from 1 to 4 h and produced an anti-inflammatory response comparable to that of standard diclofenac sodium, particularly relevant in the second phase of edema. These data suggest that *P. autumnale* may function by a similar mechanism, such as inhibiting cyclooxygenase or lipoxygenase enzymes, or by inhibiting synthesis.

The acetic acid-induced abdominal writhing test serves as a screening method for evaluating analgesic or anti-inflammatory properties in plant extracts. Acetic acid elicits localized inflammatory pain by stimulating the production of endogenous mediators that activate nociceptive neurons, which then transmit pain signals to the central nervous system and the brain via the prostaglandin pathway [47,48]. The pain induced by acetic acid is manifested in the contraction of abdominal muscles, the extension of forelimbs, and body elongation, commonly referred to as writhing, with a frequency measured over 30 min [48]. Both extracts of *P. autumnale*, particularly AAEx, demonstrated a substantial reduction in acetic acid-induced writhing in mice compared to the control group. NSAIDs mainly achieve the analgesic effect by inhibiting cyclooxygenase and/or lipoxygenase, as well as other inflammatory mediators, or by obstructing pain responses mediated by nociceptors [46]. Accordingly, *P. autumnale* extracts may have an analgesic effect via these pathways. Given that AAEx and the usual medication (paracetamol) exhibit equivalent inhibitory effects.

The phytochemical analysis highlights the high levels of polyphenols in both AgP and UgP of *P. autumnale*. Research by Megumi et al. showed that, at 50 mg/kg, apigenin and luteolin significantly reduced paw thickness by about 55%, demonstrating their effectiveness [49]. The anti-inflammatory effects of polyphenols involve inhibiting enzymes linked to inflammation, such as COX-2, LOX, and iNOS, suppressing NF-*κ*B and AP-1 activities, activating phase-II antioxidant detoxification enzymes, and stimulating pathways like MAPK, protein kinase C, and nuclear factor erythroid 2-related factor [50]. The structural differences in molecules determine whether flavonoids exhibit strong or weak bioactivities. The arrangement and number of hydroxyl groups on the flavone skeleton, along with the degree of methoxylation, significantly influence their anti-inflammatory effects. The natural flavonoid featuring two hydroxyl groups at C-5 and C-7 on the A-ring, and one at C-4′ on the B-ring, exhibits the highest anti-inflammatory activity. Generally, methoxylation enhances this activity. Moreover, the presence of a C2-C3 double bond and a 4-oxo group on the C-ring is crucial for inhibiting COX-2 transcription [49,51]. These structural features are common across many flavonoids and their derivatives, which are found in AgP and UgP, confirming their significant anti-inflammatory activity.

As mentioned before, one of the main purposes of NSAIDs is to inhibit the denaturation of proteins. Furthermore, protein denaturation is a contributing cause to inflammation. This occurs when proteins lose their biological molecule properties by changes in their tertiary and secondary structures, using external stress or by application of compounds that include a strong acid/base, concentrated inorganic salt, organic solvent, or heat, and consequently, they lose their biological role [52]. Thus, the denaturation of proteins is a widely reported factor that contributes to inflammation, and the anti-denaturation test is an important approach for assessing anti-inflammatory activity.

In the present study, the heat denaturation of BSA protein was tracked by various extracts of UgP and AgP from *P. autumnale*. Thermal denaturation is determined by temperature adjustment, which can increase the kinetic energy, inducing the rapid movement or vibration of the protein’s constituent components, causing damage to the molecule. These processes involve changes in electrostatic force and modifications in hydrogen, hydrophobic, and disulfide bonds. The denatured protein will have reduced solubility in liquid, tend to precipitate or settle, and remain irreversible [53,54]. In line with this assumption, the results provide that AAEx exhibits protein denaturation inhibitory activity, which can be seen from the IC_50_ value of 58.97 ± 1.71 µg/mL. This result is still higher than the IC_50_ of diclofenac sodium, which is 109.45 ± 1.24 µg/mL, and may be due to the ability of extract contents to protect proteins against thermal and hypotonic denaturation by protecting structural alterations in the protein.

Only 0.6% of the total African flora have been shown to have in vitro anti-inflammatory activity from 54 families, including 4.9% from the *Asparagaceae* family [55]. Most previous in vitro anti-inflammatory studies from the *Asparagaceae* family were determined by COX assays. Results of ethanolic bulb extracts from eleven *Eucomis* species exhibited moderate inhibition of COX-1 at 250 μg/mL, with no significant differences in bulb, leaf, root parts, or in specimens harvested. Madikizela B. et al. (2014) reported that the inhibition of COX-2 of leaf ethanolic extracts from *Asparagus africanus* and *Asparagus falcatus* was 50.6% and 35.2%, respectively [56]. Bulbs of *Bowiea volubilis* Harv., *Drimia elata* Jacq., and *Drimia robusta* Baker showed strong COX-1 inhibition in ethanolic extracts equivalent to 100%, 46%, and 76%, respectively, while the aqueous extracts were 73%, 8%, and 0%, respectively [57,58]. Odeyemi S. et al. (2017) used BSA denaturation as another model test to evaluate the anti-inflammatory activity of *Albuca bracteata* and *Albuca setosa,* with the IC_50_ mg/mL of methanolic extract being 0.747 and 0.555, respectively, whereas the aqueous extracts were 1.234 and 0.885, respectively [59]. Moreover, a few studies on the *Scilla* genus have been evaluated, with COX-1 inhibition of the bulb ethanolic extract reported at 81% and the aqueous extract at 11% from *Scilla natalensis* [58]. Unfortunately, the current literature does not provide any data about the anti-inflammatory activities of the *Prospero* genus in vitro or in vivo. Therefore, the present study reports the first anti-inflammatory properties of *P. autumnale* extracts, which confirms the traditional uses concerning pain and rheumatic fever.

Neurodegeneration is the progressive and sluggish loss of the functionality of particular populations of neurons and neural stem cells in specific areas of the brain, leading to sensory and motor deficiencies as well as cognitive impairment [60]. It is an essential pathological characteristic of several neurodegenerative disorders, including Alzheimer’s disease (AD) [61]. Alzheimer’s disease is responsible for around 60% of dementia cases [62], and according to the WHO, around 30 million people are affected by AD globally, with projections showing a rise to 100 million by 2050 [63].

The gradual degeneration of cholinergic neurons in the hippocampus and cortical areas of the brain is related to a significant decrease in the levels of the important neurotransmitter acetylcholine (ACh). Consequently, cholinesterase inhibitors (ChEIs) represent the basis of treatment that focuses on the cholinergic hypothesis of AD by inhibiting the metabolism of acetylcholine by acetylcholinesterase (AChE), thereby increasing its accessibility in the synaptic cleft and stimulating cholinergic receptors [64].

The three common cholinesterase inhibitors (ChEIs) are donepezil (Aricept^®^), rivastigmine (Exelon^®^), and galantamine (Razadyne^®^). However, the main disadvantages of these medications are their moderate and ephemeral benefits, which last a maximum of 12 to 24 months, and their limited efficacy since they reduce Alzheimer’s disease symptoms but do not stop disease development. Some other drawbacks include gastrointestinal disturbances and cardiovascular symptoms such as bradycardia and neuropsychiatric symptoms [62,65]. Hence, there is a need to develop novel AChE inhibitor drugs based on natural products, with the expectation of less side effects and greater selectivity. Notably, 15% of all AChE investigations carried out in the past decade belong to plant sciences [62]. However, there is no data through AD concerning *Prospero* species.

From the result of the present study (Table 5), the strong AChE inhibition of AAEx (IC_50_ µg/mL: 11.97 ± 0.62) followed by UMEx (IC_50_ µg/mL: 25.98 ± 0.57) compared with the drug galantamine (IC_50_ µg/mL: 6.27 ± 1.15) has demonstrated that *P. autumnale* may contain important compounds that can improve neurotransmission. Numerous studies indicate that phytochemicals have been identified as efficient inhibitors of AChE, primarily comprising alkaloids, cannabinoids, curcuminoids, stilbenes, and flavonoids. Among them, many flavonoids have garnered increasing attention due to their significant inhibitory efficacy and minimal toxicity [66]. Moreover, numerous investigations have shown that flavonoids, including kaempferol, quercitrin, myricetin, apigenin, and luteolin, which were identified in *P. autumnale* via LC-MS/MS, have substantial inhibitory activity with an IC_50_ ranging from 3.05 to 19.1 μM [67]. The investigation of the AChEI potential of the homoisoflavonoid (IC_50_ µM: 0.2203) isolated from *Scilla nevorsa,* compared to eserine (IC_50_ µM: 0.2201), exhibits an interesting drug candidate. Additionally, a synthetic homoisoflavonoid from a benzylidene-4-chromanone type has excellent AChEI (IC_50_ µM = 0.00249), better than donepezil (IC_50_ µM = 0.0226) [63,68]. The anti-acetylcholinesterase activity of phenolic compounds within flavonoids shows they act as non-competitive inhibitors that bind to peripheral anionic sites, mainly characterized by the residues Tyr70, Asp74, Try121, Trp279, and Tyr334 [69]. However, the structural activity relationship (SAR) indicates that the optimal AChEI activity of most flavonoids is attributed to the existence and position of the hydroxyl (OH) group on rings A and B, as well as the unsaturation of ring C [67]. AChE inhibition often increases with the rise in affinities of flavonoids, dependent on the direct interaction between the flavonoid and the active site through the formation of robust hydrogen bonds [66].

Another report in the *Asparagaceae* family showed that *Asparagus adscendens* contains conypododiol (IC_50_ µM = 2.17), a potent AChE inhibitor, and according to an in vivo test for root extract, it gave significantly reduced memory dysfunction [70]. On the other hand, an in vitro study of the methanolic extract of *Asparagus racemosus* demonstrated substantial inhibition of the AChE enzyme (IC_50_ mg/mL = 12.35 ± 1.16) [71], which is less than UMEx of *P. autumnale*, indicating its potential utility in Alzheimer’s pathology.

Neuropathologically, AD is characterized by the formation of amyloid plaques, neuritic plaques, and neurofibrillary tangles. These plaques result from the misfolding, denaturation, and accumulation of the protein of amyloid beta (Aβ) and abnormal levels of tau protein, leading to the pathological formation of extracellular senile plaques and intracellular neurofibrillary tangles (NFTs) [61,72]. Furthermore, microglial activation as a main factor of neuroinflammation is closely linked to the pathogenesis of AD. Microglia get activated in response to pathogens, abnormal stimulation, tissue damage, neurotoxins, infection, or injury. In the context of AD, microglia offer neuroprotection by removing Aβ through phagocytosis. However, when microglia lose their protective functions, inflammation, synaptic loss, and neuronal damage ensue. Moreover, the failure to clear aggregated Aβ is associated with the activation of pro-inflammatory signaling pathways [73,74]. Another factor is the increase in the neurotoxicity of Aβ by the presence of AChE [61]. All these features can be regarded as significant biomarkers in AD pathology. Under these circumstances, the importance of the anti-inflammatory activity of *P. autumnale*, specifically AAEx, which also exhibits strong AChE inhibition, indicates the possibility of containing important compounds that could inhibit pro-inflammatory mediators caused by microglia activation and would be an effective therapeutic approach in order to mitigate the progression of neurodegenerative diseases. Among the detected compounds, sulfuretin has been shown to confer protection against Aβ-induced neurotoxicity and drug-induced cell death [75]. Additional research indicates that the prolonged administration of ferulic acid protected mice from Aβ1-42-induced toxicity in vivo, and the dosages of 5.3/16 mg/kg over a duration of 6 months decreased expression of amyloid deposition in a rat model [42,76]. A notable feature in homoisoflavonoids, such as deoxysappanone B, significantly inhibited the release of neuroinflammatory mediators from BV-2 microglia, including NO, PGE2, TNF-α, IL-6, and reactive oxygen species [77].

Other therapeutic strategies concentrate on potential factors contributing to neuronal demise in AD, including oxidative stress, free radical toxicity, and, as mentioned before, neuroinflammation. Several studies have demonstrated the correlation between oxidative stress and inflammation. Reactive oxygen species (ROS) produced in brain tissues can influence both synaptic and non-synaptic communication among neurons, leading to neuroinflammation, cell death, neurodegeneration, and loss of memory [50]. The central nervous system (CNS) is especially susceptible to oxidative stress owing to its significant oxygen consumption (20% of total body consumption), high levels of polyunsaturated peroxidizable fatty acids, and an insufficient antioxidant system [78]. The strong inhibition of different *P. autumnale* extracts to the various free radicals may play an important role in scavenging and preventing the formation of ROS in neuronal cells. Moreover, studies have shown that high concentrations of bio-metals, such as Fe^2+^, in AD-affected brains can promote Aβ neurotoxicity, as well as lead to oxidative stress and the formation of reactive oxygen species (ROS), including H_2_O_2_ [63]. The high scavenging of O_2_^−^ radicals and the strong Fe^2+^ chelating of the *P. autumnale* extract, and especially AAEx and UMEx, could minimize the formation of free radicals and their toxicity in the brain.

On the other hand, monoamine oxidases (MAOs), located in the outer mitochondrial membrane, are responsible for regulating and metabolizing monoamine neurotransmitters in the brain and peripheral tissues, serving as the main source of reactive oxygen species (ROS) production in the brain. Furthermore, the increase in MAO-B activity correlates with gliosis, potentially leading to increased levels of H_2_O_2_ and oxidative free radicals [73,79]. Bioactive polyphenols may decrease these processes by directly neutralizing free radical species, preventing ‘pro-oxidant’ enzymes, activating antioxidant enzymes, or influencing the apoptotic pathway [60]. Furthermore, numerous studies have demonstrated that homoisoflavonoids and their synthesized analogs exhibit strong affinities for Aβ aggregates and have been confirmed as potent and selective inhibitors of MAO-B, as well as dual inhibitors of AChE and MAO-B, as a promising “one-compound-multi-target” approach. Among them, (*E*)-5,7-dihydroxy 3-(4-hydroxybenzylidene)chroman-4-one shows strong MAO-B inhibition IC_50_ = 13.03 nM, as well as the ability to cross the blood–brain barrier and localize inside the brain [79,80,81,82]. For this reason, *P. autumnale* components could be potential candidates as MAO-B inhibitors and novel AD drugs, owing to their modulation of neurotransmitters and ability to prevent oxidative damage in the central nervous system (CNS).

The findings of the current research demonstrate that the significance of compound content in extracts of *P. autumnale* is particularly evident in AAEx and UMEx. These extracts not only enhance cholinergic activity but may also offer neuroprotection against protein aggregation, neuroinflammation, and oxidative stress. Such observations support the traditional applications of *P. autumnale* in the treatment of neurological disorders.

The spectrum of natural sunlight contains the majority of the electromagnetic spectrum, involving 50% infrared (IR) light, 40% visible light (VIS), and 10% ultraviolet (UV) light. Humans perceive infrared radiation as heat and visible radiation optically, although ultraviolet radiation is not directly detectable.

Ultraviolet light can be categorized into three wavelength ranges: UVC (200–280 nm), UVB (280–320 nm), and UVA (320–400 nm).

UVC radiation is nearly entirely absorbed by the ozone layer, whereas UVA (95%) and UVB (5%) radiation reach the Earth in significant amounts [11,83]. Moreover, UV exposure has health advantages, including the synthesis of cholecalciferol (vitamin D3), which plays a role in immune system functions and bone metabolism and may also increase mood through endorphin release. Despite the skin’s complex defense system, comprising both enzymatic and non-enzymatic elements that protect against harmful biological effects, excessive UV radiation exposure overwhelms and decreases this cutaneous defense, resulting in cellular damage and the emergence of various skin disorders [84,85]. UVB is 1000 times more effective in inducing sunburn than UVA and is primarily responsible for several skin disorders, including immunosuppression and both non-melanoma and melanoma skin malignancies, functioning as a tumor initiator, promoter, and co-carcinogen [11].

Skin protection is an effective strategy against damage caused by ultraviolet radiation through the topical application of sunscreens, which modify the body’s response to sunlight. These products contain active components; inorganic compounds can scatter and reflect UV radiation, while organic compounds absorb UV sunlight and reflect chemical energy into light or heat. However, because of their possible toxicity in humans associated with allergic and irritative reactions, toxicological consequences as endocrine disruptors, and many ecological hazards, the use of artificial sunscreens is limited [11,86]. For this reason, natural products from *P. autumnale* may be an important source for research in new active compounds with few side effects.

The effectiveness of *P. autumnale* extracts as sunscreen is indicated by the sun protection factor (SPF); a higher SPF denotes greater protection against sunburn. According to the results, the use of various solvents in which sunscreens are dissolved can improve or decrease UV absorption, which may influence the assessment of SPF values. Based on the Commission Recommendation of the European Communities, 2006/647/EC [87], DcEx, EaEx, BEx, and UMEx of the AgP and UgP parts had a high protection with high UVB absorption. The high SPF value of these extracts may be due to their high TPC and TFC, which have strong UV absorption in comparison to the other extracts. The significance of the SPF of UEaEx is probably due to its total concentration of sunscreen substance being higher than that of the other extracts.

Nevertheless, the majority of natural polyphenols are pigments, generally red, yellow, or purple, and have the ability to absorb the UV range [84]. If phytochemical components absorb photon energy, electrons can be transferred from occupied orbitals to vacant orbitals, leading to the production of excited botanical species. This excitation initiates in the singlet state and transitions to the excited triplet state by spin inversion through the intersystem transfer of the absorbed energy. The orbital contribution to absorbance indicates the electronic transitions in polyphenols between π-type molecular orbitals throughout the molecular backbone. The UV absorption profile of polyphenols is dependent on the number and existence of aromatic rings, cyclic or conjugated double bonds, as well as the stereochemistry and type of substituents [88,89,90]. Soluble and insoluble polyphenols absorb sunlight at wavelengths of 304–350 nm and 352–385 nm, respectively. Moreover, flavonoids display an absorption wavelength range (Band II) approximately between 240 and 290 nm. Flavonoids possessing conjugations between the B and C rings exhibit an additional absorption band (Band I) in the range from around 300 to 550 nm [85]. This includes homoisoflavonoids, which demonstrate absorption at 222–230 nm and 270–297 nm for saturated systems, and between 300–367 nm for unsaturated systems [91].

In addition to reactive oxygen species generated by biological systems or sunscreen photodegradation, polyphenols can produce ROS by reacting with themselves or with some other suitable molecule in biological systems. This polyphenol phototoxicity occurs when it transitions from an excited state to the ground state, while exerting its photoprotective benefits. Consequently, the absorbed energy generates excited singlet oxygen, which is phototoxic to membrane proteins, lipids, and DNA [90]. Another important natural constituent of *P. autumnale* extracts is their antioxidant activity that can prevent phototoxicity. Given the SPF and antioxidant activity data, the different high-SPF extracts may be related to their strong ability to inhibit different free radicals, exhibiting spatially higher superoxide radical scavenging.

The effectiveness of *P. autumnale* extracts as natural sunscreen may be due to the fact that they contain a variety of natural substances, typically provide a full spectrum of UV wavelength protection, and act as natural antioxidants.

## 4. Conclusions

The present study highlights, for the first time, the ability of *P. autumnale* extracts to effectively inhibit oxidative stress through free radical scavenging and to reduce inflammation by preventing protein denaturation or inhibiting enzymes. It also underscores their potential to delay Alzheimer’s disease with AChEIs and to provide photoprotection against UV-induced skin damage. The moderate acute toxicity observed emphasizes the necessity for cautious therapeutic application and warrants additional toxicological investigations. The phytochemical analysis reveals the presence of homoisoflavonoids, a rare class of flavonoids, in the Algerian *P. autumnale*, similar to the compounds identified in a related Turkish plant, including a stilbene, a homoisoflavonoid derivative, a homoisoflavonoid dimer, and a homoisoflavone−stilbene heterodimer. This shared presence of homoisoflavonoids suggests potential for broader applications in medicinal chemistry. These important results underscore the need for further studies to isolate and chemically characterize these bioactive constituents, which are present in varying amounts in the extracts, and to deepen understanding of their mechanisms of action, with the aim of identifying new, more effective, and safer potential agents against these health problems.

## 5. Materials and Methods

### 5.1. Plant Material and Extract Preparation

Both the AgP and UgP of *P. autumnale* were collected from Setif, Algeria, in September 2021, with the necessary permissions from local authorities, and they were identified by Dr. Halis Youcef at the Center for Scientific and Technical Research. This species has been documented as a medicinal plant at http://www.theplantlist.org/ and https://www.worldfloraonline.org/ (accessed on 10 February 2026). A voucher specimen (No. 202109Bo/ProAu) was deposited in the herbarium of the VPRS laboratory at the Department of Chemistry, Kasdi Merbah University, Ouargla, Algeria. The air-dried and powdered AgP and UgP parts were treated with solvents to obtain extracts of different polarities as follows:

A quantity of 1 g from AgP and UgP was macerated in an 80/20 (*v*/*v*) methanol/H_2_O mixture for 24 h. After filtration, the crude extracts were evaporated at 40 °C to yield a hydro-methanolic extract (AHMEx, UHMEx). A quantity of 150 g of AgP and UgP was macerated once with petroleum ether at room temperature. The solutions were then concentrated under a rotavapor at 35 °C to produce the (APeEx, UPeEx) fraction. Subsequently, the dried parts were macerated three times in a methanol–water mixture (80/20, *v*/*v*). The powdered crude extracts were dissolved in warm H_2_O to prepare AAPh and UAPh, which were fractionated by liquid–liquid partition using solvents of increasing polarity, starting with CH_2_Cl_2_ (ADcEx, UDcEx), followed by ethyl acetate (AEaEx, UEaEx) and n-butanol (ABEx, UBEx). Furthermore, the undissolved residue in H_2_O from the UgP was dissolved in methanol to obtain the methanolic extract (UMEx). One gram of the two plant parts was decocted in water to prepare the aqueous extracts (AAEx, UAEx). All extracts were preserved for subsequent phytochemical, in vitro, and in vivo studies.

### 5.2. Phytochemical Content Analysis

#### 5.2.1. LC-MS/MS

The hydro-methanolic extract (HMEx) of the AgP and UgP parts of *P. autumnale* was subjected to LC-ESI-HR-MS analysis for phytochemical investigation. An Ultimate 3000 Ultra-High Pressure Liquid Chromatography (UPLC) machine, coupled to an Orbitrap Q-Exactive Classic Mass Spectrometer (ThermoFisher Scientific, Bremen, Germany), was used for LC-ESI-HR-MS/MS analysis. Chromatography was performed on a Luna Omega Polar C18 column (150 mm × 2.1 mm, 3 µm) (Phenomenex, Torrance, CA, USA) using a linear gradient program from 5% B to 95% B over 30 min at a flow rate of 200 µL/min. (A: H_2_O + 0.1% formic acid; B: CH_3_CN + 0.1%). Capillary temperature 250 °C and sheath and auxiliary gas flow (N_2_) 40 and 5 (arbitrary units) were the ESI source parameters. MS spectra were collected in both positive and negative ion modes using a full-range acquisition covering *m*/*z* 150–1500 with a resolving power of 70,000. The following settings were used in a data-dependent scan experiment to obtain the HRMS/MS product ions: AGC target, 1 × 10^5^; loop count, 4; resolution, 17,500; maximum IT, 80 ms; normalization collision energy, 30%; and isolation width, 1.6.

The mass error (Δppm) between the experimentally measured and theoretical *m/z* values were calculated as follows:

Mass error (ppm) = [(*m*/*z* observed − *m/z* theoretical)/*m*/*z* theoretical] × 10^6^

Metabolites were identified using Compound Discoverer 3.3.1 Software (ThermoFisher Scientific, Bremen, Germany).

#### 5.2.2. Total Phenolic Content Estimation

The Folin–Ciocalteu method has been used to determine the total phenolic content. Müller et al. (2010) method mixed 20 µL of extract, 100 µL of diluted FCR (1:10), and 80 µL of Na_2_CO_3_ (7.5%). After incubating for 2 h, the mixture was detected at 765 nm [92]. The total phenol concentration was measured via a gallic acid standard curve, which ranged from 25 to 200 µg/mL. The total phenol content was quantified by determining the amount of gallic acid equivalent in µg/mg of the extract.

#### 5.2.3. Total Flavonoid Content Estimation

Based on Topçu et al. (2007) with slight changes, a mixture of 50 µL of extract and 130 µL of MeOH was combined with 10 µL of CH_3_COOK (1 M) and 10 µL of (Al (NO_3_)_2_, 9H_2_O) 10% [93]. The absorbance was measured at a wavelength of 415 nm after 40 min. A calibration curve was established using a standard quercetin solution with concentrations ranging from 25 to 200 µg/mL. The results were quantified as quercetin equivalents, defined as µg QE/mg extract.

### 5.3. In Vitro Activities

#### 5.3.1. Antioxidant Activities

##### ABTS Radical Scavenging Assay

The assay by Re et al. (1999) aimed to investigate the radical-scavenging capabilities of UgP and AgP extracts, involving multiple modifications [94]. A mixture containing 19.2 mg (7 mM) of ABTS, 5 mL of H_2_O, and 3.3 mg (2.45 mM) of K_2_S_2_O_8_ was prepared. This solution was used to create the ABTS radical cation, which was then stored in the dark at room temperature overnight before being utilized. In summary, a total of 10 min was dedicated to incubating a mixture of 160 µL of ABTS^+^ and 40 µL of either an extract or a standard at different concentrations ranging from 12.5 to 800 g/mL [94]. The absorbance at 734 nm was measured using a 96-well microplate reader (Perkin Elmer, EnSpire, Singapore), with a similar blank as reference. BHT and BHA served as reference compounds in all experiments, which were conducted in triplicate. The ABTS% scavenging activity was determined using the following equation:

(1) $$RSA\left[\%\right]=\left(1-\left(\frac{As}{Ac}\right)\times 100\right),$$

A_S_ and A_c_ are the absorbance of the sample and control, respectively.

##### DPPH Radical Scavenging Assay

The inhibitory effect of AgP and UgP extracts on the 2,2-diphenyl-1-picrylhydrazyl free radical was assessed using the method described by Blois et al. (1958) [95]. In brief, 40 µL of tested extract (12.5, 25, 50…, 800 g/mL) was added to a 96-well microplate, to which 160 µL of DPPH^.^ (0.04 mg/mL) was added. After incubation for 30 min, the measurement of the discoloration at 517 nm was obtained. α-tocopherol, BHA, and BHT were used as the reference compounds, and all determinations were carried out in triplicate. The % of scavenging activity of the extracts was calculated using Equation (1) above.

##### Superoxide Radical Scavenging Activity (NBT)

Rao et al. (1990) utilized alkaline DMSO to quantify the superoxide radical scavenging activity [96]. The experiment utilized a combination of 40 μL of extract, 130 μL of alkaline DMSO (prepared by dissolving 20 mg of NaOH in 1 mL of water and then adding DMSO to make a total volume of 100 mL), and 30 μL of NBT (a solution containing 1 mg/mL of nitro blue tetrazolium in H_2_O) [96]. Tannic acid and α-tocopherol were used as reference substances to measure the absorbance at 560 nm using a 96-well microplate reader (Perkin Elmer, EnSpire, Singapore). The scavenging activity of the extracts was determined using Equation (1) provided above.

##### Reducing Power (FRAP)

The reducing power activity of the different extracts from the UgP and AgP parts of *P. autumnale* was estimated using the Oyaizu et al. (1986) method, which involved a mixture of 10 μL extract, 40 μL phosphate buffer (pH 6.6), and 50 μL potassium ferricyanide (1%) K_3_Fe(CN)_6_, which was incubated at 50 °C [97]. After 20 min of incubation, 50 μL trichloroacetic acid (TCA) (10%), 40 μL H_2_O, and 10 μL ferric chloride FeCl_3_ (0.1%) were added to the mixture and then measured at 700 nm by a 96-well microplate reader (Perkin Elmer, EnSpire, Singapore). The results are presented as micromolar equivalents of Fe^2+^ compared to a standard antioxidant.

#### 5.3.2. Anti-Inflammatory Activity (Bovine Serum Albumin Denaturation)

The anti-denaturation property of AgP and UgP extracts as anti-inflammatory agents was estimated using the method described by Kandikattu et al. (2013) with some modifications [98]. A mixture of 0.5 mL of each extract concentration or diclofenac as a standard and 0.5 mL of BSA (0.2% in Tris Buffer pH 6.8) were incubated at 37 °C for 15 min, and they were then placed in a water bath at 72 °C. After 5 min, the mixture was cooled, and the precipitated protein (protein denatured) was measured at 660 nm by a spectrophotometer. The inhibition % of protein BSA denaturation was calculated using Equation (2):

(2) $$Inhibition\;[\%]=(1-\left(\frac{Absorbance\;of\;treated\;sample}{Absorbance\;of\;control}\right)\times \;100),$$

#### 5.3.3. Acetylcholinesterase Inhibition (Alzheimer’s Disease)

The acetylcholinesterase (AChE) inhibitory activity of *P. autumnale* was assessed using colorimetric techniques with a 96-well microplate reader, following Ellman’s method (1961) [99]. In summary, 150 µL of sodium phosphate buffer (100 mM, pH 8.0) and 10 µL of either the extract test or galantamine (as a standard) were combined with 20 µL of AChE (5.32 × 10^−3^ U). Following incubation at 25 °C for 15 min, 10 µL of DTNB (0.5 mM) and 10 µL of acetylthiocholine iodide (0.71 mM) were added to the mixture for measurement at 412 nm over 15 min. The % AChEI was evaluated in comparison with the control (Ethanol with phosphate buffer pH 8) using Equation (3):

(3) $$AChEI\;[\%]=\left(\frac{E-S}{E}\right)\times 100,$$

Here, E represents the enzyme’s activity without the extract, and S represents the enzyme’s activity with the extract tested.

#### 5.3.4. Photoprotection Activity (Sun Protection Factor)

SPF values have established a worldwide standard for assessing the efficacy of sunscreen lotions. They indicate the duration a person could stay in sunlight without developing sunburn [100]. The in vitro Solar Protection Factor of several *P. autumnale* extracts were determined by the spectrophotometric method developed by Mansur et al. (1986) [101]. The absorbance of 2 mg/mL of each extract from the two parts has been evaluated in the UV-B wavelength range (290–320 nm), with 5 nm increments using a 96-well microplate reader (Perkin Elmer, EnSpire, Singapore), and three measurements were conducted at each interval. The SPF was calculated using the Mansur Equation (4):

(4) $${\mathrm{SPF}\;}_{\mathrm{spectrophotometric}}\;=\;\mathrm{CF}\;\times \sum_{290}^{320}EE\left(\lambda \right)\times I\left(\lambda \right)\times Abs(\lambda ),$$

where FC = correction factor (10), EE = erythrogenic effect, I = solar intensity spectrum, and Abs = absorbance of the sample at wavelength. The values of EE (λ) × I(λ) are constant, as stated by Sayre et al. (1979) [102].

The European Commission’s 2006 recommendations specify the sun protection factor values that correspond to the protective categories [87].

### 5.4. In Vivo Activities

#### 5.4.1. Animal Preparation

Two sexes of Swiss Albino mice (Mus musculus) (20–30 days old, 20–25 g) were used in the study, which were procured from the Pasteur Institute in Algeria (Elevage Center, Kouba, Algeria). The animals were housed in cages under typical conditions at the ASSB faculty pet shop (FSB/USTHB), with a temperature range of 20–24 °C, 12 h of light daily, and humidity levels of 50–65%; mice had full access to water and standard rodent chow for 16 h before the studies. All in vivo studies were performed according to the established recommendations of the National Research Council (NRC, 1996), Washington, DC, USA. All efforts have been made to reduce the number of animals utilized. The University Animal Experimentation Ethics Committee accepted the experimental protocols (Approval Ref No. CEEF-USTHB-08-2023/11118).

#### 5.4.2. Oral Acute Toxicity

The acute oral toxicity investigation of the UAEx and AAEx extracts of *P. autumnale* was performed using the methodology outlined in OECD Guideline 423. Nulliparous, non-pregnant female albino mice were utilized. The animals were randomly assigned according to the extract type and administered dose. For each extract, four dose levels were tested: 200, 300, 400, and 500 mg/kg body weight. Each dose group included four mice. A vehicle-treated control group was included for comparison. Thus, the experimental design consisted of control animals and two extract-treated groups, UAEx and AAEx, each evaluated at four dose levels. The aqueous extracts or vehicle were administered once by oral gavage. For 14 days following treatment, all experimental animals were examined individually daily for changes in general behavior, body weight, adverse symptoms, and mortality. After completion of the experimental period, all animals were weighed and anesthetized with a combination of ketamine (80 mg/kg) and xylazine (10 mg/kg) by intraperitoneal injection before being sacrificed by cervical dislocation. After that, the organs were extracted under the same conditions for necropsy. The LD_50_ was subsequently established.

#### 5.4.3. Acute Inflammation (Carrageenan-Induced Paw Edema)

The carrageenan-induced paw edema model was employed to evaluate the anti-inflammatory efficacy of *P. autumnale* extracts, as described by Levy (1996) [103]. Mice have been divided into four groups (*n* = 3). One group functioned as a negative control, administered 0.5 mL of saline solution orally. Three groups received infusions: the aqueous extract of each UAEx and AAEx part, as well as a reference group, all administered a dosage of 100 mg/kg b. w. 0.05 mL of a freshly produced 1% carrageenan solution in saline solution injected intradermally into the plantar part of the right hind paw of the mice one hour following the oral administration of treatments. The volume of paw edema was quantified using a Plethysmometer at 1, 2, 3, 4, and 5 h after carrageenan administration. The animals were sacrificed through cervical dislocation, and their posterior legs were cut at the tarsal joint before weighing them immediately. The left hind paw was used as a reference non-inflamed paw for comparative analysis. The mean percentage increase in paw volume with time has been calculated and compared with the control group. The anti-inflammatory activity was determined as the percentage inhibition of edema using Equation (5):

(5) $$Inhibition\%=\left[\frac{PEC-PET}{PEC}\right]\times 100,$$

%EC represents the percentage of edema in the control group (negative control), while %ET denotes the percentage of edema in the examination group (diclofenac and *P. autumnale* extracts).

The edema percentage (O%) is determined using Equation (6):

(6) $$O(\%)=\left[\frac{M\left(RPW\right)-M\left(LPW\right)}{M\left(LPW\right)}\right]\times 100,$$

M (RPW) indicates the average weight of the right paw for each group, whereas M (LPW) indicates the average weight of the left paw for each group.

#### 5.4.4. Analgesic Activity

The analgesic efficacy of *P. autumnale* extract parts was assessed utilizing the acetic acid-induced writhing test, as reported by Vogel (2002) [104]. This experiment utilized four groups, each consisting of three mice (*n* = 3). Two groups received an oral infusion dose of 0.5 mL, with UAEx and AAEx, each at 100 mg/kg of body weight. Two further groups, used as positive and negative controls, were administered paracetamol at a dosage of 100 mg/Kg body weight and saline solution, respectively. Thirty minutes following treatment, mice received an intraperitoneal injection of 0.5 mL of 0.6% acetic acid to elicit writhing behavior. The number of abdominal contractions was subsequently recorded over 15 min. The percentage of protection against abdominal writhing was utilized to evaluate the analgesic efficacy and was determined using the Equation (7):

(7) $$Protection(\%)=\left[\frac{Wc-Wt}{Wc}\right]\times 100,$$

Here, Wt indicates the mean value of writhes in the treatment groups, while Wc indicates the mean value of writhes in the control groups.

### 5.5. Statistical Analysis

Statistical analyses were performed using IBM SPSS Statistics software (version 26.0, IBM Corp., Armonk, NY, USA). Two-way analysis of variance was used to assess the main effects of the studied factors and their interaction. For variables measured repeatedly across experimental conditions, repeated-measures analysis of variance was performed. Results are shown as the mean ± SD from three measurements. Differences were considered statistically significant at *p* < 0.05.

## Figures and Tables

**Table 1:** Metabolites identified with Compound Discoverer 3.3.1 software in the AgP and UgP parts of *P. autumnale* extracts analyzed by LC-ESI-HR-MS/MS. Δ ppm indicates the mass error between the measured and theoretical *m*/*z* values.

N°	Rt	[M-H]^−^	Δppm	Formula	MS/MS	Identity	AgP	UgP
1	1.65	173.1036	−4.6	C_6_H_14_N_4_O_2_		arginine	x	x
2	1.83	341.1086	2.44	C_12_H_22_O_11_	179.06/119.03/89.02	sucrose	x	x
3	2.75	191.0190	2.09	C_6_H_8_O_7_	59.02/	citric acid	x	x
4	6.04	164.0709	4.81	C_9_H_11_NO_2_	147.04/72.01	phenilalanin	x	x
5	9.8	193.0500	−2.85	C_10_H_10_O_4_	131.03/113.02/72.99	ferulic acid	x	x
6	10.81	551.1776	2.4	C_26_H_32_O_13_	341.10/193.05/165.05	glehlinoside C	x	x
7	10.9	609.1464	0.62	C_27_H_30_O_16_	447.09/285.04	kaempferol di-glucoside	x	-
8	12.19	401.1816	2.4	C_19_H_30_O_9_	239.90/365.95	unknown	x	x
9	12.74	285.0395	−3.12	C_15_H_10_O_6_	151.00/175.04	luteolin	x	x
10	12.97	203.0820	−2.79	C_11_H_12_N_2_O_2_	157.09/141.09	tryptophan	x	x
11	13.32	593.1517	1.22	C_27_H_30_O_15_	269.05/299.06	vicenin 2	x	x
12	13.81	445.0773	−0.47	C_21_H_18_O_11_	269.05/175.02/113.02	norwogonin 8-glucuronide	x	x
13	13.92	505.0983	−0.57	C_23_H_22_O_13_	329.07/299.02/113.02	quercetin acetylglucoside	x	x
14	14.20	491.1197	0.51	C_23_H_24_O_12_	329.07/315.05/207.14	rhamnetin 3-glucoside	x	x
15	14.85	285.0765	2.7	C_16_H_14_O_5_	165.02//119.05	5,7-dihydroxy-3-(4-hydroxybenzyl)chroman-4-one	x	x
16	14.97	401.0877	3.4	C_20_H_18_O_9_	269.05	cerarvensin	x	x
17	15.03	287.0562	4.6	C_15_H_12_O_6_	259.06/125.02/201.06	dihydrokaempferol	x	x
18	14.2	477.1044	1.34	C_22_H_22_O_12_	301.04/315.05	6-methoxyluteolin 7-glucoside	x	x
19	14.36	345.0981	3.6	C_18_H_18_ O_7_	209.01/152.98/224.03	homoisoflavone	-	x
20	15.75	331.0822	2.9	C_17_H_15_O_7_	225.04/210.02	muscomin	x	x
21	16.16	361.0932	0.86	C_18_H_18_O_8_	239.06/224.03	5,7,3′-trihydroxy-6,4′,5′-trimethoxyflavanone	x	x
22	16.43	315.0510	4.7	C_16_H_12_O_7_	300.03	quercetin 3-methyl ether	x	x
23	16.58	285.0401	3.7	C_15_H_10_O_6_	217.05/199.04	kaempferol	x	x
24	16.91	337.1081	0.02	C_20_H_18_O_5_	307.06/151.03	flavanone derivative	x	x
25	17.92	269.0451	3.5	C_15_H_10_O_5_	151.00/201.06	genistein	x	x
26	18.08	329.0667	2.9	C_17_H_14_O_7_	171.10/139.11/211.13/229.14	(*E*)-3-(3,4-dihydroxybenzylidene)-5,7-dihydroxy-6-methoxychroman-4-one	x	x
27	18.11	269.0453	3.34	C_15_H_10_O_5_	151.00	apigenin	x	x
28	18.2	299.0561	2.6	C_16_H_12_O_6_	227.04	(*E*)-3-(3,4-dihydroxybenzylidene)-5,7-dihydroxychroman-4-one	x	x
29	18.63	1249.5846	−0.44	C_59_H_94_O_28_	779.42/941.48/1103.53/467.38	triterpenoid glycoside	xx	x
30	18.63	315.0873	−0.13	C_17_H_16_O_6_	209.04/195.02	5,7-dihydroxy-3-(4-hydroxybenzyl)-8-methoxychroman-4-one	x	x
31	18.70	341.0665	−0.25	C_18_H_14_O_7_	297.21/279.	apigenin -lactate	x	x
32	18.71	283.0611	3.5	C_16_H_11_O_5_	177.02	5,7-dihydroxy-3-(4-hydroxybenzylidene) chroman-4-one (4′-demethyleucomine)	x	x
33	18.81	329.0661	−1.68	C_15_H_10_O_5_	314.04/299.02	sulfuretin	x	x
34	19.51	1221.59058	0.57	C_58_H_94_O_27_	751.43/913.48/1075.53/619.39	triterpenoid glycoside	xx	x
35	19.61	343.0823	0.03	C_18_H_16_O_7_	171.10/225.05	5,8,4′-trihydroxy-3,7-dimethoxy-6-methylflavone	x	x

AgP: Areal part; UgP: underground part; “x” indicates compounds detected at comparable levels in both extracts; “xx” indicates compounds with at least a two-fold higher LC–ESI–MS signal (peak area or intensity) in one extract compared to the other, under identical analytical conditions.

**Table 2:** Total phenolic and flavonoid contents of *P. autumnale* extracts.

Extracts	TPC µg GAE/mg ex			TFC µg QE/mg ex		
	AgP	UgP		AgP	UgP	
Aqueous extract (AEx)	66.941 ± 1.018	5.47 ± 0.588	^b^	42.430 ± 0.147	19.930 ± 2.651	^a^
Petroleum ether extract (PeEx)	10.274 ± 2.668	1.352 ± 1.282	^a^	13.263 ± 5.745	38.680 ± 5.008	^a^
Methanolic extract (MEx)	NT	519.392 ± 21.503	^f^	NT	314.652 ± 20.771	^e^
Aqueous phase extract (APh)	95.862 ± 6.868	101.941 ± 6.739	^c^	49.027 ± 4.861	48.958 ± 3.977	^a^
Dichloromethane extract (DcEx)	413.215 ± 15.636	650.274 ± 14.899	^f^	231.944 ± 52.738	263.402 ± 47.287	^d^
Ethyl acetate extract (EaEx)	159.49 ± 20.211 ^c^	571.254 ± 9.622 ^c^	^e^	117.152 ± 5.303	295.138 ± 3.240	^c^
Butanol extract (BEx)	167.137 ± 17.554	357.725 ± 36.476	^d^	167.708 ± 44.488	102.430 ± 7.954	^b^

Note. Values are expressed as the mean ± standard deviation, *n* = 3. TPC is expressed as μg gallic acid equivalents/mg of extract (μg GAE/mg). TFC is expressed as μg quercetin equivalents/mg of extract (μg QE/mg). NT: not tested. Different superscript within the same parameter indicate significant differences at *p* < 0.05. Values sharing the same letter are not significantly different.

**Table 3:** IC_50_ and A_0.5_ values of AgP and UgP extracts of *P. autumnale* in different antioxidant assays.

Extracts	IC_50_ µg/mL						A_0.5_ µg/mL	
	ABTS		DPPH		NBT		FRAP	
	AgP	UgP	AgP	UgP	AgP	UgP	AgP	UgP
AEx	243.63 ± 3.55 ^c^	652.93 ± 13.66 ^b^	354.61 ± 40.65 ^cd^	1740.66 ± 49.32 ^a^	23.03 ± 3.89 ^d^	78.70± 16.38 ^bc^	34.94± 2.31 ^g^	212.31 ± 16.91 ^c^
PeEx	649.97 ± 15.33 ^b^	4804.37 ± 32.11 ^a^	NT	NT	104.91 ± 18.55 ^a^	56.55 ± 4.52 ^c^	186.60 ± 1.73 ^cd^	791.39 ± 59.07 ^a^
MEx	NT	16.28 ± 0.6 ^f^	NT	140.01 ± 4.50 ^fg^	NT	8.97 ± 0.78 ^d^	NT	22.66 ± 0.53 ^g^
APh	160.16 ± 5.69 ^d^	252.86 ± 12.84 ^c^	346.31 ± 4.44 ^cde^	NT	63.77 ± 4.92 ^bc^	89.61 ± 2.33 ^ab^	22.97 ± 0.20 ^g^	353.73 ± 44.40 ^b^
DcEx	36.19 ± 0.83 ^f^	2.86 ± 0.71 ^f^	1385.70 ± 66.01 ^b^	133.92 ± 3.47 ^g^	20.16 ± 3.57 ^d^	16.85 ± 0.49 ^d^	346.34 ± 29.25 ^b^	147.60 ± 1.79 ^cde^
EaEx	97.09 ± 2.87 ^e^	26.11 ± 0.81 ^f^	385.66 ± 57.88 ^c^	53.02 ± 1.01 ^g^	27.20 ± 1.79 ^d^	9.84 ± 0.60 ^d^	109.34 ± 5.43 ^ef^	166.66 ± 2.53 ^cde^
BEx	92.6 ± 3.39 ^e^	72 ± 0.55 ^e^	246.24 ± 5.39 ^ef^	251.42 ± 46.21 ^de^	24.03 ± 2.74 ^d^	11.15 ± 0.21 ^d^	139.91 ± 2.03 ^de^	47.93 ± 8.50 ^fg^
BHT *	1.29 ± 0.30		12.99 ± 0.41		NT		NT	
BHA *	1.81 ± 0.10		6.14 ± 0.41		NT		NT	
α-Tocopherol *	NT		13.02 ± 5.17		˂3.125		34.93 ± 2.38	
Tannic acid *	NT		NT		˂3.125		NT	
Ascorbic acid *	NT		NT		NT		6.77 ± 1.15	

* Standard compounds. IC_50_ and A_0.5_ are the concentrations corresponding to 50% inhibition and 0.50 absorbance, respectively. Values are expressed as the mean ± SD. Different superscript letters within the same assay indicate significant differences between extract × plant-part combinations at *p* < 0.05. Comparisons were not made across different assays. Standard antioxidants were used as references and were not included in the factorial ANOVA. NT: not tested. Values reported as < were not included in ANOVA.

**Table 4:** Anti-inflammatory IC_50_ of *P. autumnale* extracts.

Extract	UAEx	AAEx	AAPh	Diclofenac *
IC_50_ (µg/mL)	441.56 ± 15.26 ^a^	58.97 ± 1.71 ^b^	412.11 ± 7.68 ^c^	109.45 ± 1.24 ^d^

* Standard compounds. IC_50_ refers to the concentration required to achieve 50% inhibition. Values marked with different superscripts (a, b, c, d) are statistically significantly different (*p* < 0.05).

**Table 5:** Acetylcholinesterase inhibitory activity IC_50_ of *P. autumnale* extracts.

Extract	APeEx	AAEx	AAPh	UPeEx	UAEx	UMEx	Galantamine *
IC_50_ (µg/mL)	NA	11.97 ± 0.62 ^a^	NA	NA	NA	25.98 ± 0.57 ^b^	6.27 ± 1.15 ^c^

* Standard compounds. NA: not active. IC_50_ refers to the concentration needed to achieve 50% inhibition. Values marked with different superscripts (a, b, c) show significant differences (*p* < 0.05).

**Table 6:** In vitro sun protection factor (SPF) of *P. autumnale* extracts determined by the spectrophotometric method.

Extract	AgP		UgP	
	SPF	Protection	SPF	Protection
AEx	28.97 ± 0.81 ^b^	Medium	9.36 ± 0.03 ^b^	Low
PeEx	14.17 ± 1.31 ^a^	Low	8.24 ± 0.17 ^a^	Low
MEx	NT		42.27 ± 0.57 ^d^	High
APh	32.75 ± 0.99 ^c^	High	19.83 ± 0.71 ^c^	Medium
DcEx	NT		43.37 ± 0.17 ^c^	High
EaEx	42.89 ± 2.72 ^d^	High	44.01 ± 1.60 ^d^	High
BEx	40.97 ± 0.97 ^d^	High	43.01 ± 1.38 ^d^	High

Values are expressed as the mean ± standard deviation. Different superscript letters within the same column indicate significant differences between extracts. NT: not tested.

**Table 7:** Mortality and body weight changes in mice treated with *P. autumnale* extracts during the 14-day acute toxicity assay.

Dose mg/kg	Group	Mortality	Initial Body Weight (g)	Final Body Weight (g)
200	Control	0/4	24.73 ± 0.8	25.11 ± 0.7
	UAEx	1/4	20.43 ± 1.3 **	20.87 ± 1.0 **
	AAEx	0/4	21.38 ± 0.9 **	22.09 ± 1.0 **
300	Control	0/4	21.16 ± 0.4	22.07 ± 0.6
	UAEx	2/4	23.17 ± 0.5 ***	24.38 ± 0.9
	AAEx	0/4	24.26 ± 1.2 *	25.10 ± 1.0 **
400	Control	0/4	25.04 ± 1.1	25.11 ± 0.5
	UAEx	3/4	NT	NT
	AAEx	2/4	24.11 ± 1.2	25.34 ± 1.1
500	Control	0/4	NT	NT
	UAEx	4/4	NT	NT
	AAEx	4/4	NT	NT

Values are expressed as the mean ± SD. Initial body weight was calculated using the animals present at the start of the experiment. Final body weight was calculated using surviving animals only. For each dose level, one parallel control group was used as the common comparator for both UAEx and AAEx. Statistical comparisons were performed only between each extract-treated group and its corresponding dose-level control group. ns: not significant; * *p* < 0.05; ** *p* < 0.01; *** *p* < 0.001 versus the corresponding dose-level control. NT: not tested.

**Table 8:** Weight of vital organs in different doses of UAEx and AAEx.

Weight (g)	Control	200 mg/kg		300 mg/kg		400 mg/kg	
		UAEx	AAEx	UAEx	AAEx	UAEx	AAEx
Liver	1.10 ± 1.2 ^a^	1.15 ±0.3 ^b^	1.08 ±0.9 ^c^	1.34 ±1.3 ^d^	1.21 ±1.5 ^e^	ND	1.49 ±0.6 ^f^
Heart	0.14 ± 0.9 ^a^	0.18 ±0.2 ^b^	0.16 ±0.7 ^c^	0.13 ±1.0 ^d^	0.16 ±0.7 ^e^	ND	0.17 ±1.2 ^f^
Kidney	0.36 ± 0.5 ^a^	0.39 ±0.8 ^b^	0.34 ±0.9 ^c^	0.32 ±1.3 ^d^	0.31 ±1.1 ^e^	ND	0.36 ±0.7 ^f^

ND: not detected. The values with different superscripts (a, b, c, d, e, f) in the same lines are significantly different (*p* < 0.05).

**Table 9:** Anti-inflammatory effect of *P. autumnale* extracts on carrageenan-induced paw edema.

Treatment	Dose (mg/kg)	% Edema	% Inhibition
Control	-	59	-
Diclofenac	100	18.31	68.96
AAEx		19.53	66.89
UAEx		21.07	64.28

*n* = 3 mice per group. Diclofenac was used as a reference anti-inflammatory drug. Paw edema percentage and inhibition percentage were calculated relative to the control group. (-) Not applicable: no inhibition value was calculated for the control group.

**Table 10:** Analgesic effect of *P. autumnale* extracts in the acetic acid-induced writhing test.

Treatment	Dose (mg/kg)	No. of Writhing	% Writhing Inhibition
Control	-	77	-
Paracetamol	100	27.14	64.75
AAEx		27.23	64.63
UAEx		30.81	59.98

*n* = 3 mice per group. Paracetamol was used as the reference analgesic drug. Writhing inhibition was calculated relative to the control group. (-) No inhibition value was calculated for the control group.

## Data Availability

Data are contained within the article.

## Abbreviations

The following abbreviations are used in this manuscript:

AgP	Aerial ground part
UgP	Underground part
A	Aerial
U	Under
AEx	Aqueous extract
HMEx	Hydro-methanolic extract
PeEx	Petroleum ether extract
APh	Aqueous phase
MEx	Methanolic extract
DcEx	Dichloromethane extract
EaEx	Ethyl acetate extract
BEx	Butanol extract
